# Adenosine deaminase for diagnosis of tuberculous pleural effusion: A systematic review and meta-analysis

**DOI:** 10.1371/journal.pone.0213728

**Published:** 2019-03-26

**Authors:** Ashutosh Nath Aggarwal, Ritesh Agarwal, Inderpaul Singh Sehgal, Sahajal Dhooria

**Affiliations:** Department of Pulmonary Medicine, Postgraduate Institute of Medical Education and Research, Chandigarh, India; University of Texas MD Anderson Cancer Center, UNITED STATES

## Abstract

**Objective:**

Pleural fluid adenosine deaminase (ADA) is a useful diagnostic test for tuberculous pleural effusion (TPE), but its exact threshold and accuracy in clinical decision-making is unclear. We aimed to assess diagnostic performance of ADA in TPE and to clarify its optimal diagnostic threshold.

**Methods:**

We searched PubMed, Embase, and Cochrane Library databases for articles indexed up to October 2018. We included English language studies that provided both sensitivity and specificity of ADA in TPE diagnosis. Summary estimates for sensitivity and specificity were obtained through bivariate random effects model, both overall and at prespecified threshold ranges of <36, 40±4, 45–65 and >65 IU/L.

**Results:**

We retrieved 2162 citations, and included 174 publications with 27009 patients. All studies showed high risk of bias. Summary sensitivity, specificity and diagnostic odds ratio estimates were 0.92 (95% CI 0.90–0.93), 0.90 (95% CI 0.88–0.91) and 97.42 (95% CI 74.90–126.72) respectively. 65 studies with ADA threshold of 40±4 IU/L showed summary sensitivity and specificity of 0.93 (95% CI 0.90–0.95) and 0.90 (95% CI 0.87–0.91) respectively. Four studies with ADA threshold >65 IU/L showed summary sensitivity and specificity of 0.86 (95% CI 0.61–0.96) and 0.94 (95% CI 0.80–0.99) respectively.

**Conclusion:**

ADA levels in pleural fluid show good diagnostic accuracy in diagnosis of TPE; however, all included studies showed high risk of bias. It was not possible to derive any firm inference on relative clinical utility of different diagnostic thresholds.

## Introduction

Tuberculosis (TB) remains a major cause of morbidity and mortality throughout the world. The World Health Organization (WHO) estimated nearly 10.4 million new cases globally in 2015, with maximum burden in the developing world [1]. Pulmonary TB is the commonest form of TB, and a definite microbiological diagnosis is possible in most instances through sputum microscopy, culture and the use of cartridge-based nucleic acid amplification tests (CBNAAT). Extrapulmonary TB, representing nearly 15% of the global TB burden, is more difficult to diagnose. Tuberculous pleural effusion (TPE), one of the commonest forms of extrapulmonary TB, is a diagnostic challenge with rather poor microbiologic confirmation rates from pleural fluid analysis [2, 3]. Even diagnostic tools like CBNAAT and interferon-gamma release assays have shown suboptimal diagnostic accuracy [4, 5].

Adenosine deaminase (ADA), an enzyme produced from lymphocytes and involved in purine metabolism, has been extensively studied as a biochemical marker in pleural fluid during investigation for TPE. The test is simple, cheap, rapid, minimally invasive, and can be performed in most laboratories [3]. Although pleural fluid ADA is not a perfect discriminator, its level is considerably elevated in patients with TPE. High ADA levels can sometimes be observed in pleural fluid from patients of empyema, malignancy, or rheumatoid pleurisy [6]. Therefore, presence of raised pleural fluid ADA is considered a useful marker for diagnosis of TPE, especially in patients with exudative and lymphocytic pleural effusion in high TB burden settings. These patients can empirically be started on anti-tuberculous therapy if no other investigation can provide a definite diagnosis [7, 8]. Similarly, low pleural fluid ADA may be useful in excluding TPE, especially in a patient with low pre-test probability [6, 7]. These patients usually require additional investigations to establish the etiology of pleural effusion. Previous meta-analyses have suggested good diagnostic performance for pleural fluid ADA [9–14]. While reviewing published literature for a meta-analysis of Indian studies that we recently published, we realized that many additional studies had become available since the publication of the previously largest meta-analysis [11, 14]. Moreover, despite its widespread use, there is still no clarity on an optimum discriminatory threshold value. In general, the probability of having (or not having) TPE rises (or falls) as the pleural fluid ADA level increases (or decreases) [3]. Previous meta-analyses have failed to address this issue in detail. Both the availability of several new studies, and the need to clarify a clinically useful diagnostic threshold, highlight the necessity of performing a fresh analysis [15]. In this study, we evaluate the diagnostic accuracy of pleural fluid ADA in the diagnosis of TPE among patients with pleural effusion, and attempt to define a clinically useful test threshold value, through an updated systematic review and meta-analysis of published English literature.

## Materials and methods

We performed this review in accordance with the Preferred Reporting Items for Systematic Reviews and Meta-Analyses (PRISMA) statement [16, 17]. An Institutional Review Board approval was not necessary as this work involved systematic review and meta-analysis of previously published studies. We searched the PubMed, Embase, and Cochrane Library databases, for articles indexed up to December 2016. We updated our search on October 31, 2018. Our search strategy for the PubMed database was ("adenosine deaminase"[MeSH Terms] OR "adenosine deaminase"[All Fields] OR "ADA"[All Fields]) AND ("pleura"[MeSH Terms] OR "pleura"[All Fields] OR "pleural"[All Fields] OR "pleuritis"[All Fields] OR "pleurisy"[MeSH Terms] OR "pleurisy"[All Fields] OR "nonrespiratory"[All Fields] OR "non-respiratory"[All Fields] OR "extrapulmonary"[All Fields] OR "extra-pulmonary"[All Fields]). A similar search strategy was used for the other two databases.

Titles and abstracts of studies extracted through electronic database search were screened to find articles potentially appropriate for further detailed evaluation. Articles published in foreign languages, animal studies, and publications not primarily related to pleural TB were excluded at this stage. Full-texts of relevant publications were independently read by two reviewers (ANA and RA) to identify studies suitable for data synthesis. All disagreements were resolved through consensus. We finally included only those original articles that provided both sensitivity and specificity values for pleural fluid ADA in diagnosing TPE, or provided numerical or graphical data from which both these measures could be calculated. Biochemical, experimental, and descriptive studies were not further considered. Conference abstracts, case reports, reviews (including systematic reviews), editorials, and letters to editor not describing original diagnostic accuracy data were also excluded. For studies reporting on overlapping patient datasets, only the publication providing greater methodologic detail, or that with a larger sample size, was included. Wherever data from more than a single diagnostic threshold was reported, we selected the one providing the largest sum of sensitivity and specificity for further analysis. A diagnosis of TPE was based on one or more of following criteria: (a) microbiologic (demonstration of acid-fast bacilli on pleural fluid or biopsy smear, or culture or polymerase chain reaction positivity for *M*. *tuberculosis* in pleural or other clinical specimens); (b) pathologic (granulomatous inflammation in pleural biopsy specimen); and, (c) clinical (response to empiric antitubercular treatment). Such a composite definition of reference standard was adopted due to absence of a well-performing confirmatory test to diagnose TPE, which results in a presumptive diagnosis in several patients, including those enrolled in research studies.

Two reviewers (ANA and RA) independently extracted information from all included studies regarding publication year, country of study, study design, etiology of non-tubercular effusions, ADA assay method, ADA threshold, serologic testing for human immunodeficiency virus (HIV), percentage of TPE patients identified using only pathologic and/or microbiologic criteria (hereafter referred to as ‘definite TB’), and total positive and negative test results for each patient group. Quality of each publication was evaluated through the QUADAS-2 (Quality assessment for studies of diagnostic accuracy) tool by the same two reviewers [18]. Any disagreements were resolved through consensus.

We computed sensitivity and specificity for each study, along with corresponding 95% confidence intervals (95% CI). We anticipated a high degree of study level variability and heterogeneity, and hence pooled our data using a bivariate random effects model. We computed summary estimates for sensitivity, specificity, and diagnostic odds ratio (DOR). Positive and negative likelihood ratios (PLR and NLR) were computed from these summary estimates, and PLRs above 10, or NLRs less than 0.1, reflected the test’s ability to confirm or exclude disease respectively [19]. A hierarchical summary receiver operating characteristic (HSROC) plot was also constructed to display variability in diagnostic accuracy across publications, and summarize overall test performance [20]. Heterogeneity was expressed using *I*^*2*^ statistic and categorized as high if *I*^*2*^ was more than 75% [21]. Heterogeneity was also explored after stratifying data based on predefined covariates. These included period of publication, study design (prospective or otherwise), geographic area, TB burden in the country of study, TB prevalence in the study population (>50% or not), characteristics of non-tubercular effusions (whether exudative only or not), ADA measurement technique (Guisti or non-Guisti), ADA threshold (<36, 40±4, 45–65 or >65 IU/L), study sample size (>100 patients or not), blinding (whether explicitly stated or not), and robustness of reference standard (definite TB or composite clinical criteria). The 30 high TB burden countries were identified based on recent World Health Organization recommendations [1]. ADA threshold categories were arbitrarily selected based on general expert opinions that (a) ADA levels below 40 IU/L may rule out a diagnosis of TPE, (b) the most common diagnostic threshold is around 40 IU/L, and (c) higher ADA levels (especially above 70 IU/L or so in a lymphocytic pleural effusion) virtually establish a diagnosis of TPE [3, 8]. Heterogeneity was further evaluated through restricted maximum likelihood estimation meta-regression analysis, using the natural logarithm of DOR as the independent variable. Publication bias was assessed by Deek’s funnel plot, and a slope coefficient with p <0.10 implied significant asymmetry suggestive of bias [22].

The statistical software Stata (intercooled version 12.0, Stata Corp, College Station, TX), particularly its ‘midas’, ‘metandi’ and ‘metareg’ command packages, was used for data analysis. We added 0.5 to all cells with zero values for calculations involving logit or logarithmic transformations.

## Results

**Fig 1:**
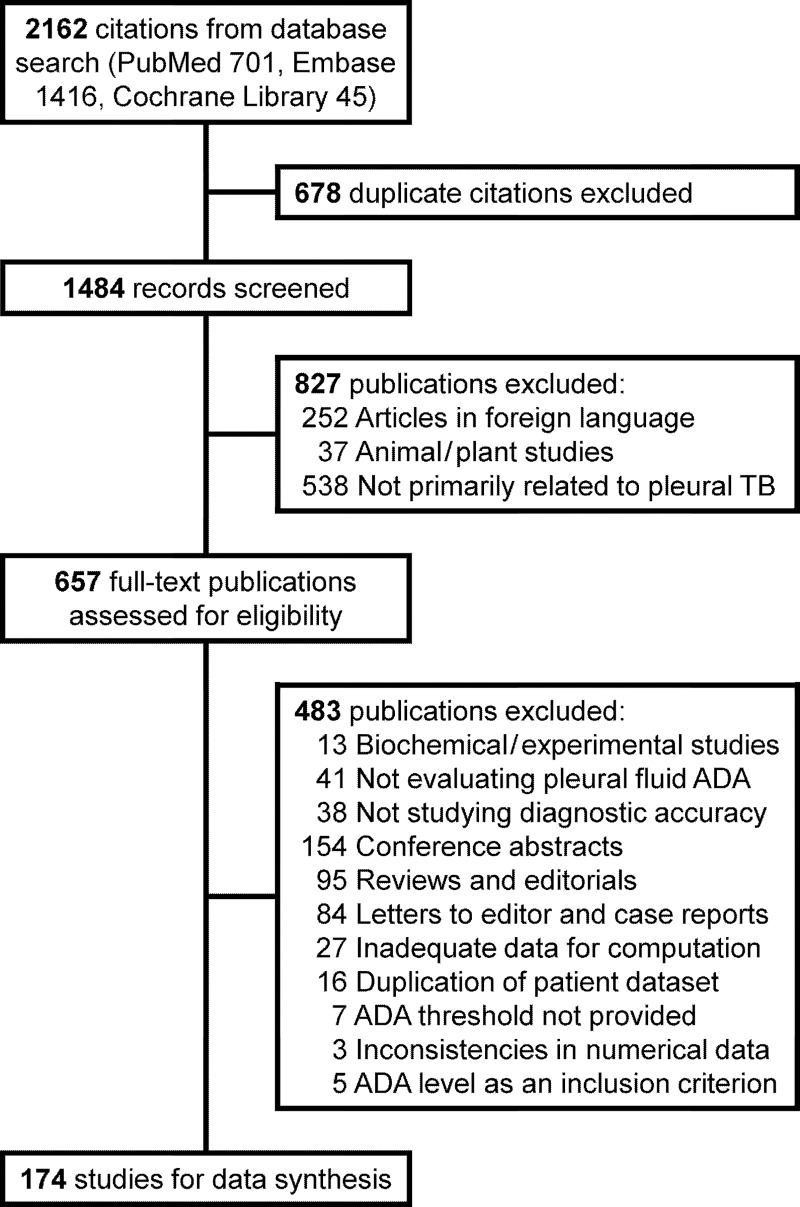
Study selection process.

We retrieved 2162 citations from our database search, and assessed 657 full-text articles after initial title and abstract review (Fig 1). We finally selected 174 studies for data synthesis (S1 Table of online supplement). Details on these individual publications are provided in S2 and S3 Tables of the online supplement. The studies finally included for review yielded information on 10696 TPE patients and 16313 patients with pleural effusions of other etiologies, giving an overall TPE prevalence of 39.6%. 84 (48.3%) studies were conducted in countries currently designated by the WHO as ‘high burden countries’. 120 (69.0%) studies had a prospective design. Blinding was ensured in 26 (14.9%) studies only. Only 16 (9.1%) studies reported on inclusion of human immunodeficiency seropositive subjects. 85 (48.9%) studies included patients with exudative effusions only. 80 (46.0%) studies used a ‘definite TB’ reference standard. 68 (39.1%) studies used composite clinical criteria for diagnosing TPE whereas 26 (14.9%) studies provided no such information. Most studies (105, 60.3%) reported ADA measurements as per the colorimetric procedure proposed by Guisti; however, the diagnostic thresholds varied widely (S3 Table, online supplement). 18 (10.3%) studies reported diagnostic accuracy data using multiple ADA thresholds. In all, we analyzed 65 (37.4%) studies using a ADA threshold from 36–44 IU/L, and another 56 (32.2%) using 45–65 IU/L as the ADA threshold. The most common threshold used in our analysis was 40 IU/L (43 studies having 2718 TPE patients and 3306 patients of other effusions). Only four (2.3%) studies provided data from thresholds of 70 IU/L or more.

Study quality assessment through the QUADAS-2 tool revealed high risk of bias for all studies (S1 Fig, online supplement). This primarily resulted from a high risk of bias for index test domain due to absence of information on blinding and/or failure to use a pre-specified ADA threshold.

**Fig 2:**
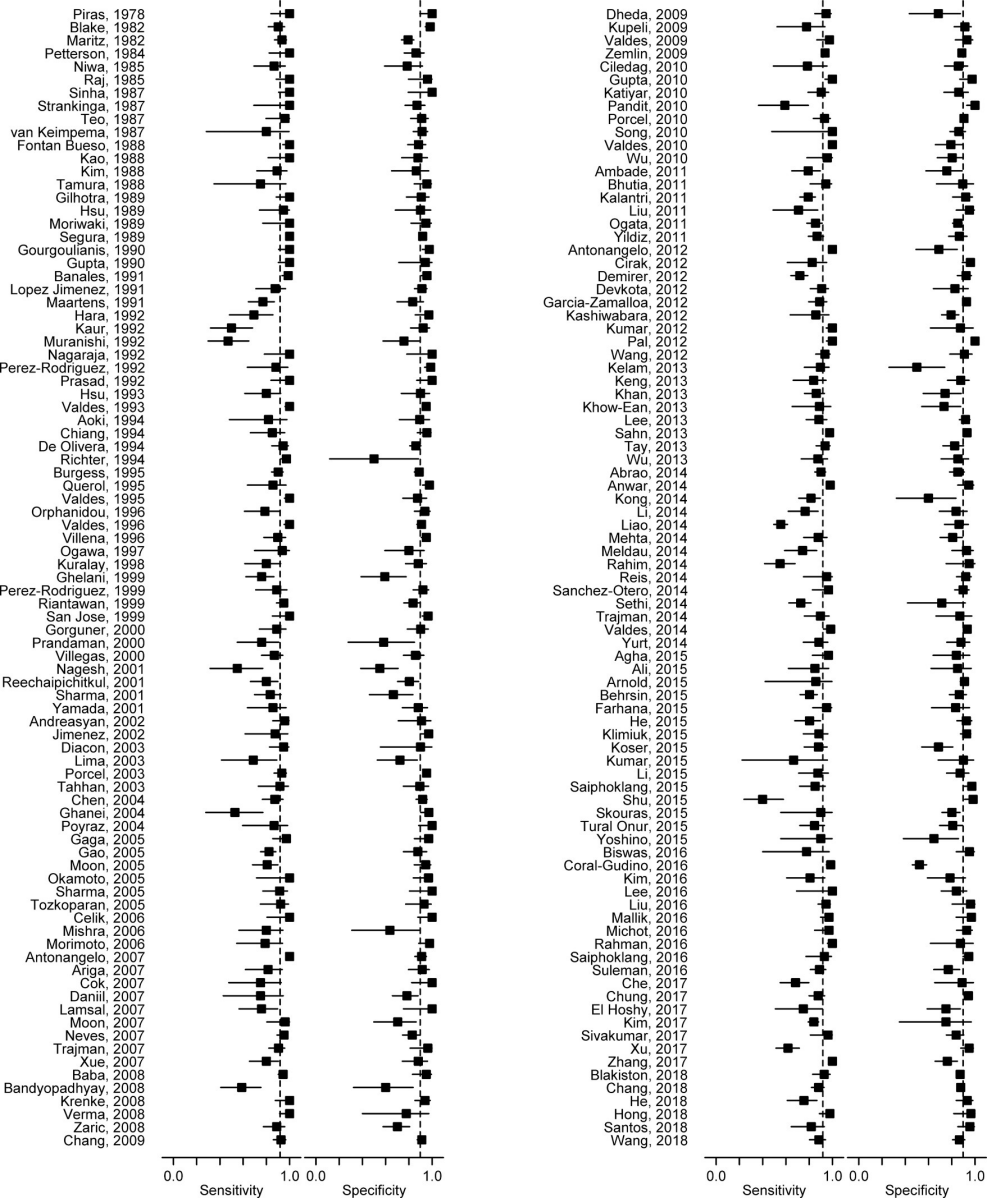
Forest plot of 174 studies on sensitivity and specificity of pleural fluid adenosine deaminase in diagnosing tuberculous pleural effusion. Solid squares indicate individual study estimates, and horizontal lines represent corresponding 95% confidence limits. Vertical dashed lines correspond to summary estimates of sensitivity and specificity.

**Fig 3:**
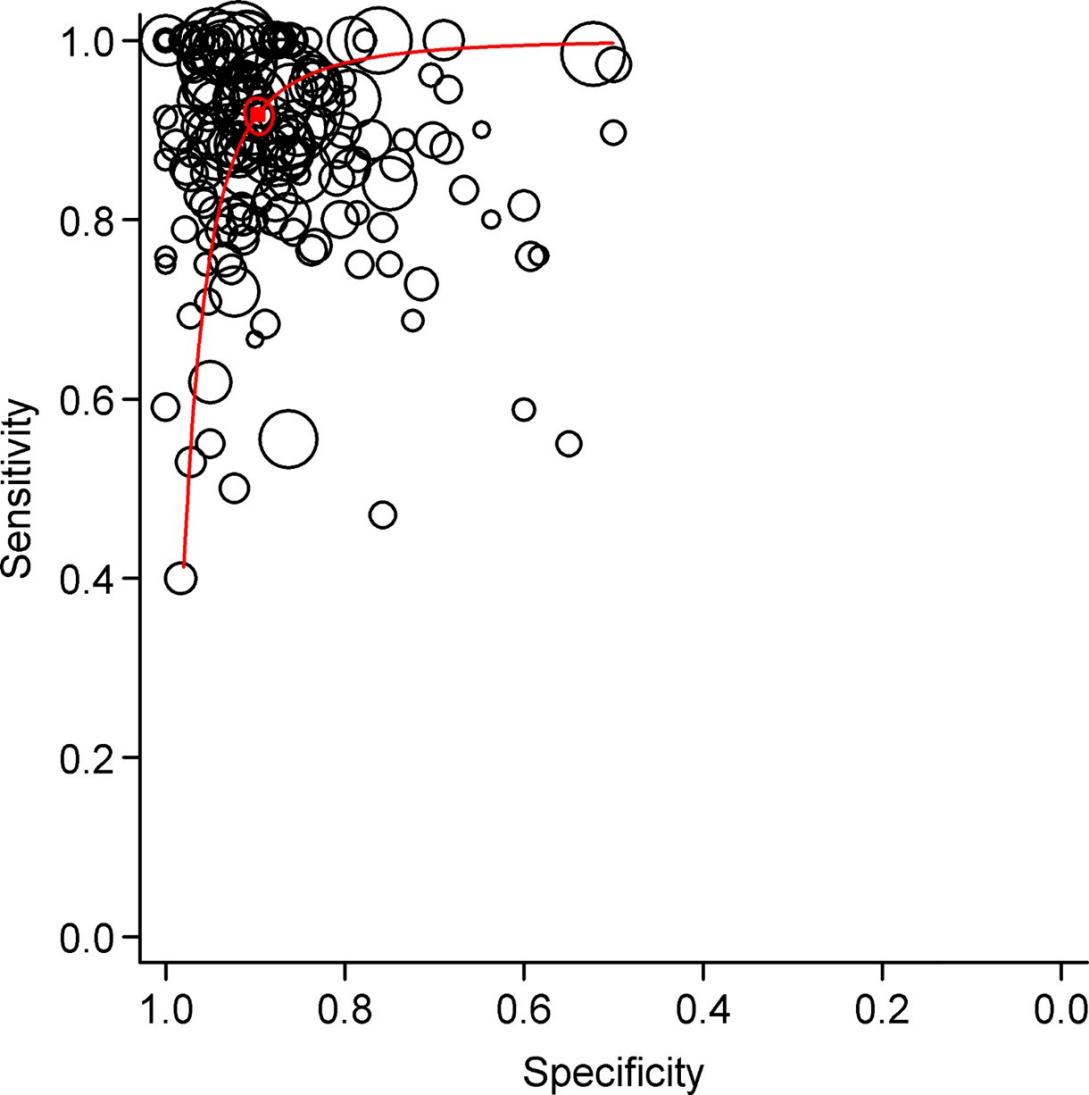
Hierarchical summary receiver operating characteristic (HSROC) curves summarizing diagnostic performance of pleural fluid adenosine deaminase for diagnosis of tuberculous pleural effusion. Each individual study is represented by an open circle, whose size is proportional to the inverse standard error of sensitivity and specificity. The dark square represents the summary estimate of test accuracy, with the surrounding dashed zone outline denoting the 95% confidence region around this estimate.

Diagnostic accuracy measures from individual studies are summarized in S4 Table of online supplement. Sensitivity figures ranged from 0.40 to 1.00 across studies (Fig 2), with a summary estimate of 0.92 (95% CI 0.90–0.93) (Table 1). Specificity figures varied from 0.50 to 1.00, with a summary estimate of 0.90 (95% CI 0.88–0.91). Summary DOR was 97.42 (95% CI 74.90–126.72). The summary sensitivity/specificity point on the HSROC curve (Fig 3) was located close to the desired upper left corner, and location and shape of curve suggested pleural fluid ADA assay to be a good discriminator.

**Table 1:** Subgroup analysis for exploration of factors influencing diagnostic performance of pleural fluid adenosine deaminase (ADA).

Parameter	Category (no. of studies)	Summary sensitivity (95% CI)	*I*^*2*^, %	Summary specificity (95% CI)	*I*^*2*^, %
Year of publication	Up to 2000 (N = 50)	0.95 (0.92–0.97)	88.22	0.91 (0.89–0.93)	76.77
	2001 to 2010 (N = 49)	0.91 (0.88–0.94)	82.70	0.90 (0.87–0.92)	78.76
	After 2010 (N = 75)	0.90 (0.87–0.92)	91.87	0.88 (0.86–0.90)	87.23
Prospective study design	Yes (N = 120)	0.91 (0.89–0.93)	90.85	0.90 (0.88–0.91)	77.73
	No, or not specified (N = 54)	0.93 (0.91–0.95)	77.99	0.90 (0.87–0.92)	90.45
Geographic area	Africa (N = 13)	0.91 (0.87–0.94)	76.73	0.87 (0.81–0.91)	76.32
	Indian subcontinent (N = 35)	0.93 (0.87–0.96)	92.36	0.90 (0.84–0.94)	87.07
	China and far east (N = 57)	0.87 (0.84–0.90)	89.04	0.89 (0.87–0.91)	69.59
	West Asia (N = 17)	0.83 (0.79–0.87)	60.31	0.91 (0.86–0.94)	72.58
	Europe (N = 38)	0.97 (0.95–0.98)	79.88	0.92 (0.89–0.93)	94.13
	South America (N = 11)	0.93 (0.85–0.97)	85.63	0.86 (0.83–0.89)	53.72
	Others (N = 3)	Not estimable	-	Not estimable	-
Country’s TB burden	High (N = 84)	0.91 (0.88–0.93)	92.12	0.89 (0.86–0.91)	78.65
	Not high (N = 90)	0.92 (0.90–0.94)	85.06	0.91 (0.89–0.92)	87.44
TB prevalence in study	>50% (N = 101)	0.92 (0.90–0.94)	85.39	0.91 (0.90–0.93)	78.51
population	< = 50% (N = 73)	0.92 (0.89–0.94)	92.44	0.87 (0.84–0.89)	85.68
Nature of effusion	Exudates only (N = 85)	0.92 (0.90–0.94)	86.91	0.88 (0.85–0.90)	85.93
	Transudates also, or not specified (N = 89)	0.91 (0.89–0.93)	91.56	0.91 (0.90–0.92)	76.33
ADA assay technique	Guisti (N = 105)	0.93 (0.90–0.95)	92.31	0.89 (0.88–0.91)	79.24
	Non-Guisti, or not specified (N = 69)	0.90 (0.88–0.92)	82.26	0.90 (0.88–0.92)	88.33
ADA threshold	<36 IU/L (N = 49)	0.91 (0.88–0.94)	91.10	0.89 (0.87–0.91)	72.84
	40±4 IU/L (N = 65)	0.93 (0.90–0.95)	90.37	0.90 (0.87–0.91)	89.13
	45–65 IU/L (N = 56)	0.91 (0.88–0.94)	87.02	0.90 (0.87–0.92)	79.36
	>65 IU/L (N = 4)	0.86 (0.61–0.96)	88.56	0.94 (0.80–0.99)	83.94
Study sample size	>100 patients (N = 80)	0.93 (0.91–0.95)	93.28	0.90 (0.88–0.91)	89.34
	< = 100 patients (N = 94)	0.90 (0.87–0.92)	82.61	0.90 (0.88–0.92)	76.16
Blinding in study	Yes (N = 26)	0.86 (0.80–0.90)	85.59	0.86 (0.82–0.89)	75.62
	No, or not specified (N = 148)	0.93 (0.91–0.94)	90.62	0.90 (0.89–0.92)	85.27
Reference standard	Definite (N = 80)	0.93 (0.90–0.95)	91.82	0.90 (0.88–0.91)	71.32
	Composite, or not specified (N = 94)	0.91 (0.89–0.93)	87.65	0.90 (0.88–0.91)	88.32

95% CI 95% confidence interval, *I*^*2*^ Heterogeneity statistic, TB Tuberculosis

There was no clear change in diagnostic performance with increasing ADA thresholds (S2 Fig, online supplement). Pooled sensitivity and specificity estimates for studies having ADA thresholds of >44 IU/L were largely similar to studies with thresholds from 36–44 IU/L (Table 1), with some suggestion of higher specificity for studies using ADA thresholds >65 IU/L. Data from these studies suggest that the more commonly used ADA thresholds around 40 IU/L had low NLR (Table 2) and may thus be more useful for excluding, rather than confirming, a diagnosis of TPE. Estimates from four studies with ADA thresholds above 65 IU/L suggested higher PLR, and higher positive predictive values across a range of TPE prevalence (Table 2). Such thresholds might thus provide better confirmation, but poorer exclusion, of TPE.

**Table 2:** Diagnostic accuracy estimates based on different thresholds of pleural fluid adenosine deaminase.

Adenosine deaminase threshold range	<36 IU/L	40±4 IU/L	45–65 IU/L	>65 IU/L	Overall
Number of studies	49	65	56	4	174
Median prevalence of tuberculosis in studies	0.39	0.46	0.41	0.44	0.41
Summary sensitivity	0.91 (0.88–0.94)	0.93 (0.90–0.95)	0.91 (0.88–0.94)	0.86 (0.61–0.96)	0.92 (0.90–0.93)
Summary specificity	0.89 (0.87–0.91)	0.90 (0.87–0.92)	0.90 (0.87–0.92)	0.94 (0.80–0.99)	0.90 (0.88–0.91)
Summary diagnostic odds ratio	86.32 (56.42–132.08)	109.51 (71.52–167.67)	91.57 (55.05–152.33)	96.55 (14.75–632.17)	97.42 (74.90–126.72)
Positive likelihood ratio*	8.60	8.88	8.91	14.62	8.92
Negative likelihood ratio*	0.10	0.08	0.10	0.15	0.09
Positive predictive value at median prevalence*	0.85	0.88	0.86	0.92	0.86
Negative predictive value at median prevalence*	0.94	0.93	0.94	0.89	0.94
Positive predictive value at 20% prevalence*	0.68	0.69	0.69	0.79	0.69
Negative predictive value at 20% prevalence*	0.98	0.98	0.98	0.96	0.98
Positive predictive value at 60% prevalence*	0.93	0.93	0.93	0.96	0.93
Negative predictive value at 60% prevalence*	0.87	0.89	0.87	0.81	0.88

Figures in parentheses are 95% confidence limits

* Values calculated from summary sensitivity and specificity estimates

There was substantial heterogeneity between studies (*I*^*2*^ 89.94% and 84.03% respectively for sensitivity and specificity). On subgroup analysis, the magnitude of change between summary estimates of sensitivity and specificity in each prespecified subgroup was small (Table 1). Multivariate meta-regression suggested that only total study sample size, and study blinding, significantly influenced relative DOR (S5 Table, online supplement).

Deeks’ funnel plot asymmetry test showed a slope coefficient of -12.06 (p<0.001) for studies included in this review. This was interpreted as asymmetry in data, suggestive of publication bias (S3 Fig, online supplement).

## Discussion

The previous largest meta-analysis on this topic included 63 studies indexed till March 2007 [11]. We have updated this by 111 additional studies, both published during the last decade as well as older studies not included in the earlier review. In addition, we also address the clinically important issue of choosing an appropriate ADA threshold during patient evaluation.

The present data analysis suggests that pleural fluid ADA has good sensitivity (0.92) and specificity (0.90) for diagnosing TPE. Based on the HSROC curve (Fig 3), ADA appears to be a good discriminator test for diagnosing TPE. However, its diagnostic utility varies considerably across different geographical regions, clinical settings and test result thresholds. These observations are largely similar to those obtained from previous meta-analyses. Our data suggests that a positive test improves post-test probability of TPE to a much lower extent in high prevalence than in low prevalence settings, and a negative test performs much better at excluding TPE in low prevalence settings (S4 Fig in online supplement). This broad interpretation, however, does not account for the variable thresholds used in different studies.

Despite four decades of research, the optimum pleural fluid ADA cut-off for the diagnosis of TPE remains unclear. In general, ADA levels higher than 70 IU/L are highly suggestive of TPE, whereas levels less than 40 IU/L are more helpful in excluding disease [3]. We decided a-priori to tailor our analysis to specifically include these threshold ranges. Overall, our data does not support significant improvement in specificity (as is generally thought) with incremental thresholds. The three groups with ADA thresholds <36, 40±4, and 45–65 IU/L yielded similar likelihood ratios (Table 1). Specifically, NLR for the 40±4 IU group suggests that there are only eight false negatives for every 100 true negatives, implying that this threshold can be used to exclude TPE. However, the PLR suggests that there are ten false positives for every 89 true positives; hence this threshold is less useful to confirm TPE. Unfortunately, our systematic review yielded only four studies using ADA threshold above 65 IU/L. PLR estimated from these studies was high, hence such threshold may be more useful in disease confirmation (only ten false positives for every 146 true positives). Our data therefore suggests that patients with low pleural fluid ADA (below 36–44 IU/L) should be evaluated in detail for a non-tubercular disorder. A pleural biopsy may be helpful in this setting, more so if other investigations like pleural fluid malignant cytology are noncontributory [23]. Although the strength of our evidence is weak, a higher level (>65 IU/L) may perhaps be more suggestive of TPE. One can consider initiating empiric anti-tubercular therapy in these patients, especially in high prevalence areas and if other laboratory and clinical parameters also support this diagnosis. However, we were constrained by availability of only a small number of studies in this area.

Our meta-analysis is not without limitations. We restricted our analysis to articles published in English language, and may have missed some clinical studies. As only 80 of 174 studies employed definite microbiologic and/or pathologic criteria to diagnose TPE, we cannot rule out a misclassification bias. We documented considerable heterogeneity between studies, and a multivariate meta-regression model still showed considerable residual variation due to heterogeneity. This might make our conclusions less robust. Further, the quality of most included studies was suboptimal. Many studies included patients with transudative effusions, which may have improved specificity estimates, even though TPE would not normally be suspected in such settings. Similarly, very few studies looked only at exudative lymphocytic effusions, which would remain the predominant scenario where TPE is suspected. We were unable to extract individual patient data for such pleural fluids and cannot comment on the diagnostic performance of ADA in relation to lymphocyte proportion in pleural fluid. Several studies reported diagnostic thresholds that optimized trade-off between sensitivity and specificity, rather than those that were clinically more relevant for confirming (or excluding) TPE. We also adopted a similar approach while selecting one among many thresholds reported by some studies. We have summarized diagnostic performance of pleural fluid ADA as a single test and cannot comment on its additive value in routine decision making, once results from other investigations are concurrently available. The predictive ability of pleural fluid ADA has been shown to improve with addition of clinical and other laboratory data [24].

In conclusion, the present meta-analysis suggests that ADA levels in pleural fluid show a good diagnostic accuracy in diagnosis of TPE. However, all included studies showed a risk of bias, and several had included patients with transudative effusions. Diagnostic accuracy in these studies was variable across geographic regions and clinical settings. The included studies used variable ADA thresholds for diagnosis, and we could not derive any firm inference on the relative utility of low or high thresholds in routine patient assessment. Large well-planned multicenter studies are required to establish clinically useful thresholds of ADA for confirming or excluding a diagnosis of TPE.

## Supporting information

S1 Fig Risk of bias and applicability concerns summary. (PDF)

S2 Fig Diagnostic accuracy as a function of pleural fluid adenosine deaminase (ADA) threshold value in various studies. (PDF)

S3 Fig Deek's funnel plot assessment test for evaluation of any potential publication bias. (PDF)

S4 Fig Bayesian conditional probability plots for pleural fluid adenosine deaminase (ADA) assay. (PDF)

S1 Table Studies included in data synthesis. (PDF)

S2 Table Characteristics of studies included in data synthesis. (PDF)

S3 Table Clinical characteristics, and adenosine deaminase assay technique and results, from studies included in data synthesis. (PDF)

S4 Table Diagnostic accuracy estimates from included studies. (PDF)

S5 Table Multivariate meta-regression analysis. (PDF)

S6 Table PRISMA checklist. (PDF)
