# Supplementary material for: Adenosine deaminase for diagnosis of tuberculous pleural effusion: A systematic review and meta-analysis
Source: PLoS One. 2019 Mar 26;14(3):e0213728. doi: 10.1371/journal.pone.0213728 (PMC6435228; doi:10.1371/journal.pone.0213728)

S1 Fig. Risk of bias and applicability concerns summary

Study	Risk of Bias: Patient Selection	Risk of Bias: Index Test	Risk of Bias: Reference Standard	Risk of Bias: Flow and Timing	Applicability Concerns: Patient Selection	Applicability Concerns: Index Test	Applicability Concerns: Reference Standard
Piras, 1978	?	–	?	+	+	+	+
Blake, 1982	+	–	?	+	–	+	+
Maritz, 1982	+	–	+	+	–	+	+
Petterson, 1984	+	–	+	+	–	+	+
Niwa, 1985	–	–	+	–	–	+	+
Raj, 1985	+	–	+	+	–	+	+
Sinha, 1987	+	–	?	–	–	+	+
Strankinga, 1987	+	–	+	–	–	+	+
Teo, 1987	+	–	+	+	–	+	+
van Keimpema, 1987	+	–	+	+	–	+	+
Fontan Bueso, 1988	–	–	+	+	–	+	+
Kao, 1988	+	–	+	+	–	–	+
Kim, 1988	?	–	–	+	–	+	+
Tamura, 1988	?	–	?	–	–	–	+
Gilhotra, 1989	+	–	+	+	–	+	+
Hsu, 1989	–	–	+	+	–	+	+
Moriwaki, 1989	–	–	+	+	+	+	+
Segura, 1989	+	–	+	+	–	+	+
Gourgoulianis, 1990	?	–	+	?	?	+	+
Gupta, 1990	–	–	+	+	+	+	+
Banales, 1991	+	–	+	+	+	+	+
Lopez Jimenez, 1991	?	–	?	+	–	+	+
Maartens, 1991	–	–	+	–	–	+	+
Hara, 1992	+	–	+	+	+	+	+
Kaur, 1992	+	–	+	+	–	+	+
Muranishi, 1992	–	–	+	+	–	+	+
Nagaraja, 1992	+	–	+	+	–	+	+
Perez-Rodriguez, 1992	+	–	?	+	–	+	+
Prasad, 1992	?	–	+	+	–	+	+
Hsu, 1993	–	–	+	+	–	+	+
Valdes, 1993	+	–	+	–	–	+	+
Aoki, 1994	+	–	+	+	–	+	+
Chiang, 1994	?	–	+	+	–	+	+
De Olivera, 1994	+	+	–	+	–	+	+
Richter, 1994	+	–	+	+	+	+	+
Burgess, 1995	+	–	+	+	–	+	+
Querol, 1995	+	–	+	+	–	+	+
Valdes, 1995	+	–	+	+	+	+	+
Orphanidou, 1996	?	–	+	+	–	+	+
Valdes, 1996	+	–	+	+	–	+	+
Villena, 1996	+	–	+	+	–	+	+
Ogawa, 1997	–	–	+	+	–	+	+
Kuralay, 1998	?	–	+	+	–	+	+
Ghelani, 1999	+	–	–	+	–	+	+
Perez-Rodriguez, 1999	+	–	+	+	–	+	+
Riantawan, 1999	+	–	?	+	–	+	+
San Jose, 1999	+	–	+	+	–	+	+
Gorguner, 2000	+	–	+	+	–	+	+
Prandaman, 2000	+	–	?	–	–	+	–
Villegas, 2000	+	–	+	+	–	+	+
Nagesh, 2001	+	–	+	+	–	+	+
Reechaipichitkul, 2001	+	–	+	+	+	+	+
Sharma, 2001	+	–	–	+	–	+	+
Yamada, 2001	?	–	+	+	–	+	+
Andreasyan, 2002	+	–	?	+	+	+	+
Jimenez, 2002	–	–	+	+	+	+	+
Diacon, 2003	+	–	+	+	+	+	+
Lima, 2003	+	–	–	+	+	+	+
Porcel, 2003	–	–	–	+	–	+	+
Tahhan, 2003	+	–	+	–	+	+	+
Chen, 2004	–	–	?	+	+	+	+
Ghanei, 2004	+	–	+	+	–	+	+
Poyraz, 2004	?	–	+	+	–	+	+
Gaga, 2005	–	–	+	+	–	+	+
Gao, 2005	–	–	?	+	–	+	+
Moon, 2005	+	–	+	+	+	+	+
Okamoto, 2005	–	–	+	+	–	+	+
Sharma, 2005	+	–	+	+	+	+	+
Tozkoparan, 2005	–	–	–	–	–	+	+
Celik, 2006	?	–	+	+	+	+	+
Mishra, 2006	+	–	–	+	+	+	+
Morimoto, 2006	?	–	?	+	–	+	+
Antonangelo, 2007	–	–	–	+	–	+	+
Ariga, 2007	–	–	+	+	–	+	+
Cok, 2007	–	–	–	+	–	+	+
Daniil, 2007	+	–	+	+	+	+	+
Lamsal, 2007	?	–	–	+	?	+	+
Moon, 2007	+	–	+	+	+	+	+
Neves, 2007	–	–	+	+	+	+	+
Trajman, 2007	–	–	+	–	–	+	+
Xue, 2007	–	–	+	+	–	+	+
Baba, 2008	–	–	+	–	–	+	+
Bandyopadhyay, 2008	–	–	?	+	–	+	+
Krenke, 2008	–	–	+	+	–	+	+
Verma, 2008	+	–	+	+	+	+	+
Zaric, 2008	–	–	+	+	–	+	+
Chang, 2009	–	–	+	+	+	+	+
Dheda, 2009	+	+	+	+	+	+	+
Kupeli, 2009	+	–	?	–	–	+	+
Valdes, 2009	+	–	+	+	+	+	+
Zemlin, 2009	–	–	+	–	–	+	+
Ciledag, 2010	?	–	+	+	–	+	+
Gupta, 2010	+	–	–	+	+	+	+
Katiyar, 2010	+	–	+	+	+	+	+
Pandit, 2010	+	–	–	+	+	+	+
Porcel, 2010	–	–	+	+	–	+	+
Song, 2010	?	–	+	?	+	+	+
Valdes, 2010	+	–	+	+	+	+	+
Wu, 2010	–	–	+	+	–	+	+
Ambade, 2011	+	–	+	–	+	+	+
Bhutia, 2011	+	–	?	+	–	+	+
Kalantri, 2011	+	–	–	+	–	+	+
Liu, 2011	–	–	+	+	–	+	+
Ogata, 2011	+	–	+	+	–	+	+
Yildiz, 2011	–	–	+	+	–	+	+
Antonangelo, 2012	–	–	?	+	–	+	+
Cirak, 2012	?	–	+	+	–	+	+
Demirer, 2012	–	–	+	+	–	+	+
Devkota, 2012	+	–	–	+	–	+	+
Garcia-Zamalloa, 2012	+	–	–	+	–	+	+
Kashiwabara, 2012	+	–	–	+	+	+	+
Kumar, 2012	+	–	–	+	+	+	–
Pal, 2012	?	–	?	+	–	+	+
Wang, 2012	+	–	+	+	–	+	+
Kelam, 2013	+	–	+	+	+	+	+
Keng, 2013	+	–	+	+	–	+	+
Khan, 2013	+	–	+	+	+	+	+
Khow-Ean, 2013	–	–	+	+	–	+	+
Lee, 2013	–	–	+	+	–	+	+
Sahn, 2013	–	–	+	+	–	+	+
Tay, 2013	–	–	+	+	+	+	+
Wu, 2013	–	–	+	+	–	+	+
Abrao, 2014	+	–	+	+	+	+	+
Anwar, 2014	+	–	?	+	–	+	+
Kong, 2014	+	–	+	+	–	+	+
Li, 2014	–	–	+	+	–	+	+
Liao, 2014	+	–	–	+	–	+	+
Mehta, 2014	+	–	?	+	+	+	+
Meldau, 2014	+	–	+	–	–	+	+
Rahim, 2014	–	–	–	+	–	+	–
Reis, 2014	+	–	?	+	+	+	+
Sanchez-Otero, 2014	?	–	?	–	+	+	+
Sethi, 2014	+	+	–	+	+	+	+
Trajman, 2014	–	–	–	–	–	+	+
Valdes, 2014	–	–	+	+	–	+	+
Yurt, 2014	–	–	+	+	+	+	+
Agha, 2015	–	–	+	+	–	+	+
Ali, 2015	–	–	+	+	–	+	+
Arnold, 2015	+	–	+	+	–	+	+
Behrsin, 2015	+	–	+	+	–	+	+
Farhana, 2015	+	–	–	+	+	+	+
He, 2015	–	–	+	+	+	+	+
Klimiuk, 2015	–	–	+	+	–	+	+
Koser, 2015	+	–	+	+	+	+	+
Kumar, 2015	+	–	+	+	+	+	+
Li, 2015	–	–	+	+	+	+	+
Saiphoklang, 2015	–	–	–	+	–	+	+
Shu, 2015	–	–	+	+	+	+	+
Skouras, 2015	–	–	+	+	+	+	+
Tural Onur, 2015	+	–	+	+	+	+	+
Yoshino, 2015	–	–	+	+	+	+	+
Biswas, 2016	+	–	+	+	–	+	–
Coral-Gudino, 2016	–	–	+	+	–	+	+
Kim, 2016	–	–	+	+	+	+	+
Lee, 2016	–	–	+	+	–	+	+
Liu, 2016	+	–	+	+	+	+	+
Mallik, 2016	+	–	–	+	+	+	+
Michot, 2016	+	–	+	+	+	+	+
Rahman, 2016	–	–	+	+	+	+	+
Saiphoklang, 2016	+	–	–	+	+	+	+
Suleman, 2016	+	–	+	+	+	+	+
Che, 2017	+	–	+	+	+	?	+
Chung, 2017	+	–	+	–	+	+	+
El Hoshy, 2017	–	–	+	+	–	+	+
Kim, 2017	–	–	+	–	–	?	+
Sivakumar, 2017	–	–	+	?	–	+	+
Xu, 2017	+	–	+	+	+	+	+
Zhang, 2017	+	–	+	–	+	+	+
Blakiston, 2018	–	–	–	?	+	+	+
Chang, 2018	+	–	+	–	–	+	+
He, 2018	–	–	+	–	+	+	+
Hong, 2018	–	–	+	+	–	+	+
Santos, 2018	–	–	+	+	+	+	+
Wang, 2018	+	–	+	+	+	+	+

+ Low – High ? Unclear

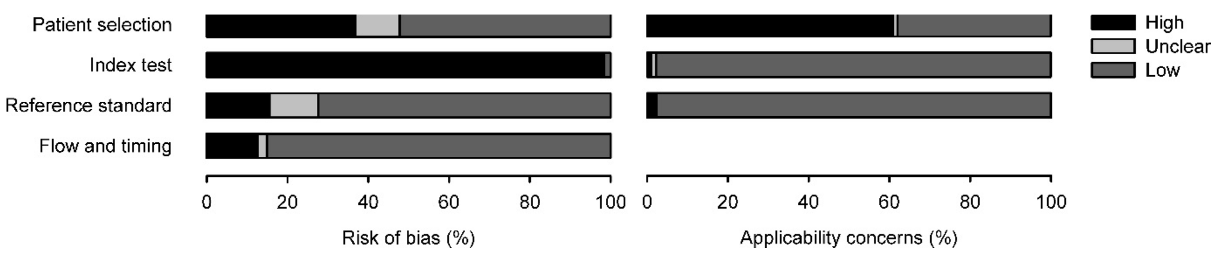

Supplement: S1 Fig — (PDF) [file pone.0213728.s001.pdf]
