# Supplementary material for: Adenosine deaminase for diagnosis of tuberculous pleural effusion: A systematic review and meta-analysis
Source: PLoS One. 2019 Mar 26;14(3):e0213728. doi: 10.1371/journal.pone.0213728 (PMC6435228; doi:10.1371/journal.pone.0213728)

S2 Fig. Diagnostic accuracy as a function of pleural fluid adenosine deaminase (ADA) threshold value in various studies. A simple linear regression line has been fitted to each scatterplot.

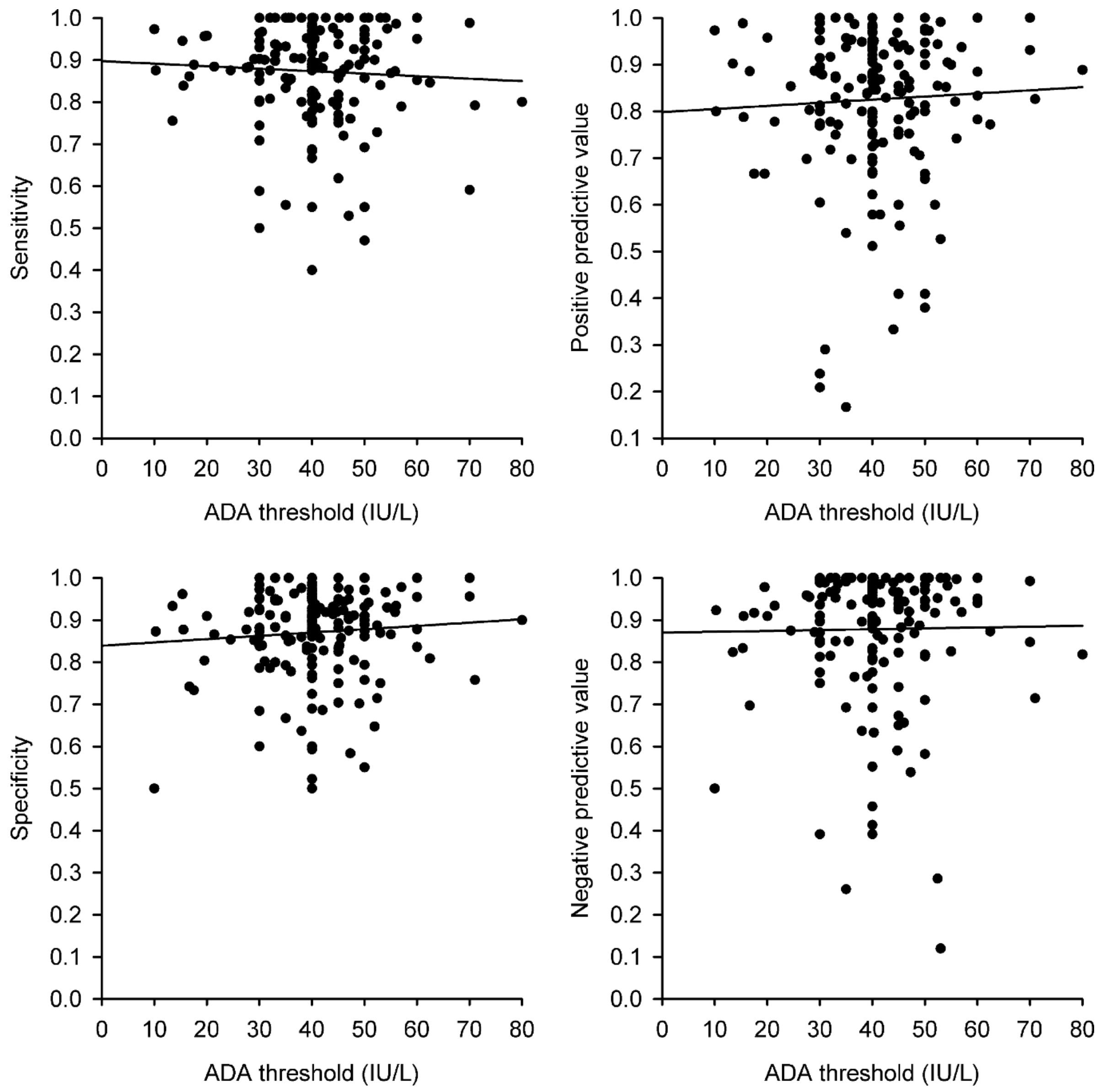

Supplement: S2 Fig — (PDF) [file pone.0213728.s002.pdf]
