# Supplementary material for: Adenosine deaminase for diagnosis of tuberculous pleural effusion: A systematic review and meta-analysis
Source: PLoS One. 2019 Mar 26;14(3):e0213728. doi: 10.1371/journal.pone.0213728 (PMC6435228; doi:10.1371/journal.pone.0213728)

**S3 Fig**. Deek's funnel plot assessment test for evaluation of any potential publication bias. This plot shows an asymmetric distribution of log of diagnostic odds ratios against inverse root of effective sample sizes, indicating presence of publication bias.

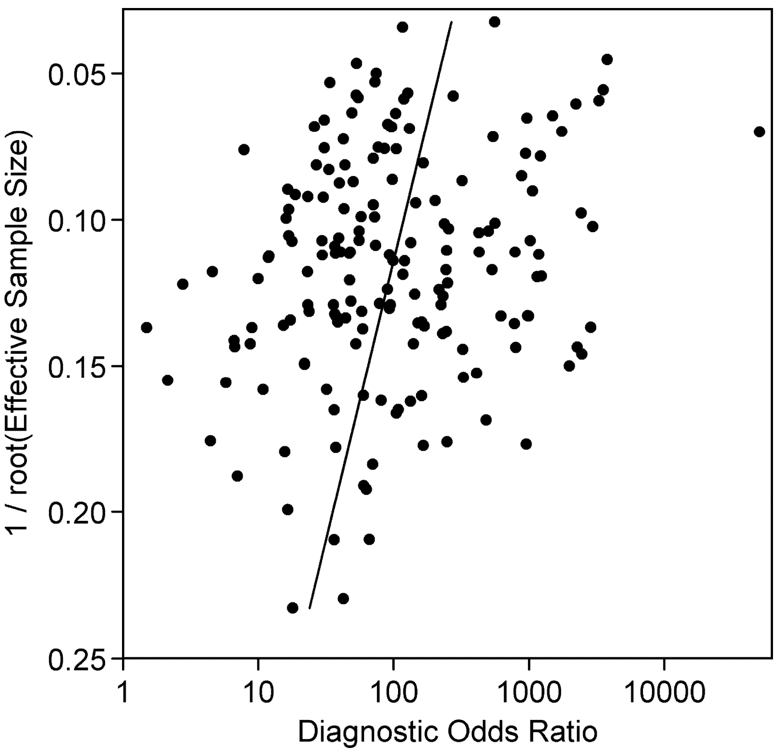

Supplement: S3 Fig — (PDF) [file pone.0213728.s003.pdf]
