# Supplementary material for: Adenosine deaminase for diagnosis of tuberculous pleural effusion: A systematic review and meta-analysis
Source: PLoS One. 2019 Mar 26;14(3):e0213728. doi: 10.1371/journal.pone.0213728 (PMC6435228; doi:10.1371/journal.pone.0213728)

S4 Fig. Bayesian conditional probability plots for pleural fluid adenosine deaminase (ADA) assay. The curves depict estimated post-test probability of tuberculous pleural effusion in a patient, given a pre-test probability of disease and a positive or negative ADA result, using summary estimates of ADA sensitivity and specificity derived during meta-analysis.

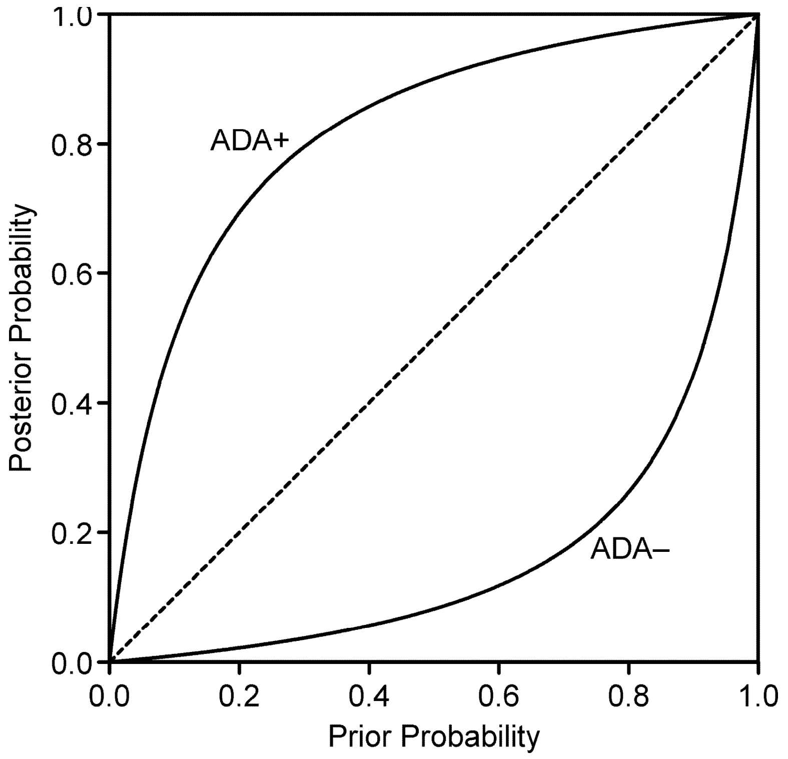

Supplement: S4 Fig — (PDF) [file pone.0213728.s004.pdf]
