# Supplementary material for: Adenosine deaminase for diagnosis of tuberculous pleural effusion: A systematic review and meta-analysis
Source: PLoS One. 2019 Mar 26;14(3):e0213728. doi: 10.1371/journal.pone.0213728 (PMC6435228; doi:10.1371/journal.pone.0213728)
Supplement: S1 Table — (PDF) [file pone.0213728.s005.pdf]

S1 Table. Studies included in data synthesis

1. Piras MA, Gakis C, Budroni M, Andreoni G. Adenosine deaminase activity in pleural effusions: an aid to differential diagnosis. Br Med J. 1978;2(6154):1751-2.
2. Blake J, Berman P. The use of adenosine deaminase assays in the diagnosis of tuberculosis. S Afr Med J. 1982;62(1):19-21.
3. Maritz FJ, Malan C, Le Roux I. Adenosine deaminase estimations in the differentiation of pleural effusions. S Afr Med J. 1982;62(16):556-8.
4. Pettersson T, Ojala K, Weber TH. Adenosine deaminase in the diagnosis of pleural effusions. Acta Med Scand. 1984;215(4):299-304.
5. Niwa Y, Kishimoto H, Shimokata K. Carcinomatous and tuberculous pleural effusions. Comparison of tumor markers. Chest. 1985;87(3):351-5.
6. Raj B, Chopra RK, Lal H, Saini AS, Singh V, Kumar P, et al. Adenosine deaminase activity in pleural fluids - a diagnostic aid in tuberculous pleural effusion. Indian J Chest Dis Allied Sci. 1985;27(2):76-80.
7. Sinha PK, Sinha BB, Sinha AR. Adenosine deaminase activity as a diagnostic index of pleural effusion. J Indian Med Assoc. 1987;85(1):11-3.
8. Strankinga WF, Nauta JJ, Straub JP, Stam J. Adenosine deaminase activity in tuberculous pleural effusions: a diagnostic test. Tubercle. 1987;68(2):137-40.
9. Teo SK, Chio LF. Adenosine deaminase in pleural fluid - an enzymatic test for tuberculous pleural effusion. Singapore Med J. 1987;28(3):220-4.
10. van Keimpema AR, Slaats EH, Wagenaar JP. Adenosine deaminase activity, not diagnostic for tuberculous pleurisy. Eur J Respir Dis. 1987;71(1):15-8.
11. Fontan Bueso J, Verea Hernando H, Garcia-Buela JP, Dominguez Juncal L, Martin Egana MT, Montero Martinez MC. Diagnostic value of simultaneous determination of pleural adenosine deaminase and pleural lysozyme/serum lysozyme ratio in pleural effusions. Chest. 1988;93(2):303-7.
12. Kao SJ, Wang D, Chang FY, Hsu K, Shen CY, Chen J. The evaluation of ADA activity in pleural effusion for the diagnosis of tuberculous pleural effusion. Kekkaku. 1988;63(6):441-6.
13. Kim JW, Yang IA, Oh EA, Rhyoo YG, Jang YH, Ryang DW, et al. C-reactive protein, sialic acid and adenosine deaminase levels in serum and pleural fluid from patients with pleural effusion. Korean J Intern Med. 1988;3(2):122-7.
14. Tamura S, Nishigaki T, Moriwaki Y, Fujioka H, Nakano T, Fujii J, et al. Tumor markers in pleural effusion diagnosis. Cancer. 1988;61(2):298-302.
15. Gilhotra R, Sehgal S, Jindal SK. Pleural biopsy and adenosine deaminase enzyme activity in effusions of different aetiologies. Lung India. 1989;7(3):122-4.
16. Hsu WH, Chiang CD, Chen WT, Chen CF. Diagnostic value of adenosine deaminase and gamma-interferon in tuberculous and malignant pleural effusions. Taiwan Yi Xue Hui Za Zhi. 1989;88(9):879-82.
17. Moriwaki Y, Kohjiro N, Itoh M, Nakatsuji Y, Okada M, Ishihara H, et al. Discrimination of tuberculous from carcinomatous pleural effusion by biochemical markers: adenosine deaminase, lysozyme, fibronectin and carcinoembryonic antigen. Jpn J Med. 1989;28(4):478-84.
18. Segura RM, Pascual C, Ocana I, Martinez-Vazquez JM, Ribera E, Ruiz I, et al. Adenosine deaminase in body fluids: a useful diagnostic tool in tuberculosis. Clin Biochem. 1989;22(2):141-8.
19. Gourgoulianis KI. Diagnostic value of adenosine deaminase activity in tuberculous effusions. Eur Respir J. 1990;3(9):1098.
20. Gupta DK, Suri JC, Goel A. Efficacy of adenosine deaminase in the diagnosis of pleural effusions. Indian J Chest Dis Allied Sci. 1990;32(4):205-8.
21. Banales JL, Pineda PR, Fitzgerald JM, Rubio H, Selman M, Salazar-Lezama M. Adenosine deaminase in the diagnosis of tuberculous pleural effusions. A report of 218 patients and review of the literature. Chest. 1991;99(2):355-7.
22. Lopez Jimenez M, Rodriguez-Pinero A, Carnicero MA, Zapatero A, Perianes J, Vigil L, et al. Adenosine deaminase in the diagnosis of pleural effusions. Adv Exp Med Biol. 1991;309b:195-8.
23. Maartens G, Bateman ED. Tuberculous pleural effusions: increased culture yield with bedside inoculation of pleural fluid and poor diagnostic value of adenosine deaminase. Thorax. 1991;46(2):96-9.
24. Hara N, Abe M, Inuzuka S, Kawarada Y, Shigematsu N. Pleural SC5b-9 in differential diagnosis of tuberculous, malignant, and other effusions. Chest. 1992;102(4):1060-4.
25. Kaur A, Basha A, Ranjan M, Oommen A. Poor diagnostic value of adenosine deaminase in pleural, peritoneal & cerebrospinal fluids in tuberculosis. Indian J Med Res. 1992;95:270-7.
26. Muranishi H, Nakashima M, Hirano H, Saitoh T, Takahashi H, Tanaka K, et al. Simultaneous measurements of adenosine deaminase activity and tuberculostearic acid in pleural effusions for the diagnosis of tuberculous pleuritis. Intern Med. 1992;31(6):752-5.

27. Nagaraja MV, Ashokan PK, Hande HM. Adenosine deaminase in pleural effusions. J Assoc Physicians India. 1992;40(3):157-9.

28. Perez Rodriguez E, Ferrando C, Flandes J. Adenosine deaminase in pleural effusion. Chest. 1992;102(1):325-6.

29. Prasad R, Tripathi RP, Mukerji PK, Singh M, Srivastava VM. Adenosine deaminase activity in pleural fluid - a diagnostic test of tuberculous pleural effusion. Indian J Chest Dis Allied Sci. 1992;34(3):123-6.

30. Hsu WH, Chiang CD, Huang PL. Diagnostic value of pleural adenosine deaminase in tuberculous effusions of immunocompromised hosts. J Formos Med Assoc. 1993;92(7):668-70.

31. Valdes L, San Jose E, Alvarez D, Sarandeses A, Pose A, Chomon B, et al. Diagnosis of tuberculous pleurisy using the biologic parameters adenosine deaminase, lysozyme, and interferon gamma. Chest. 1993;103(2):458-65.

32. Aoki Y, Katoh O, Nakanishi Y, Kuroki S, Yamada H. A comparison study of IFN-gamma, ADA, and CA125 as the diagnostic parameters in tuberculous pleuritis. Respir Med. 1994;88(2):139-43.

33. Chiang CS, Chiang CD, Lin JW, Huang PL, Chu JJ. Neopterin, soluble interleukin-2 receptor and adenosine deaminase levels in pleural effusions. Respiration. 1994;61(3):150-4.

34. De Oliveira HG, Rossatto ER, Prolla JC. Pleural fluid adenosine deaminase and lymphocyte proportion: clinical usefulness in the diagnosis of tuberculosis. Cytopathology. 1994;5(1):27-32.

35. Richter C, Perenboom R, Swai AB, Kitinya J, Mtoni I, Chande H, et al. Diagnosis of tuberculosis in patients with pleural effusion in an area of HIV infection and limited diagnostic facilities. Trop Geogr Med. 1994;46(5):293-7.

36. Burgess LJ, Maritz FJ, Le Roux I, Taljaard JJ. Use of adenosine deaminase as a diagnostic tool for tuberculous pleurisy. Thorax. 1995;50(6):672-4.

37. Querol JM, Minguez J, Garcia-Sanchez E, Farga MA, Gimeno C, Garcia-de-Lomas J. Rapid diagnosis of pleural tuberculosis by polymerase chain reaction. Am J Respir Crit Care Med. 1995;152(6 Pt 1):1977-81.

38. Valdes L, Alvarez D, San Jose E, Juanatey JR, Pose A, Valle JM, et al. Value of adenosine deaminase in the diagnosis of tuberculous pleural effusions in young patients in a region of high prevalence of tuberculosis. Thorax. 1995;50(6):600-3.

39. Orphanidou D, Gaga M, Rasidakis A, Dimakou K, Toumbis M, Latsi P, et al. Tumour necrosis factor, interleukin-1 and adenosine deaminase in tuberculous pleural effusion. Respir Med. 1996;90(2):95-8.

40. Valdes L, San Jose E, Alvarez D, Valle JM. Adenosine deaminase (ADA) isoenzyme analysis in pleural effusions: diagnostic role, and relevance to the origin of increased ADA in tuberculous pleurisy. Eur Respir J. 1996;9(4):747-51.

41. Villena V, Navarro-Gonzalvez JA, Garcia-Benayas C, Manzanos JA, Echave J, Lopez-Encuentra A, et al. Rapid automated determination of adenosine deaminase and lysozyme for differentiating tuberculous and nontuberculous pleural effusions. Clin Chem. 1996;42(2):218-21.

42. Ogawa K, Koga H, Hirakata Y, Tomono K, Tashiro T, Kohno S. Differential diagnosis of tuberculous pleurisy by measurement of cytokine concentrations in pleural effusion. Tuber Lung Dis. 1997;78(1):29-34.

43. Kuralay F, Comlekci A. Adenosine deaminase activity: to be an useful marker in distinguishing pleural effusions due to malignancy from tuberculosis. Biochem Soc Trans. 1998;26(2):S163.

44. Ghelani DR, Parikh FS, Hakim AS, Pai-Dhungat JV. Diagnostic significance of immunoglobulins and adenosine deaminase in pleural effusion. J Assoc Physicians India. 1999;47(8):787-90.

45. Perez-Rodriguez E, Perez Walton IJ, Sanchez Hernandez JJ, Pallares E, Rubi J, Jimenez Castro D, et al. ADA1/ADAp ratio in pleural tuberculosis: an excellent diagnostic parameter in pleural fluid. Respir Med. 1999;93(11):816-21.

46. Riantawan P, Chaowalit P, Wongsangiem M, Rojanaraweewong P. Diagnostic value of pleural fluid adenosine deaminase in tuberculous pleuritis with reference to HIV coinfection and a Bayesian analysis. Chest. 1999;116(1):97-103.

47. San Jose ME, Valdes L, Saavedra MJ, De Vega JM, Alvarez D, Vinuela J, et al. Lymphocyte populations in tuberculous pleural effusions. Ann Clin Biochem. 1999;36 (Pt 4):492-500.

48. Gorguner M, Cerci M, Gorguner I. Determination of adenosine deaminase activity and its isoenzymes for diagnosis of pleural effusions. Respirology. 2000;5(4):321-4.

49. Parandaman V, Narayanan S, Narayanan PR. Utility of polymerase chain reaction using two probes for rapid diagnosis of tubercular pleuritis in comparison to conventional methods. Indian J Med Res. 2000;112:47-51.

50. Villegas MV, Labrada LA, Saravia NG. Evaluation of polymerase chain reaction, adenosine deaminase, and interferon-gamma in pleural fluid for the differential diagnosis of pleural tuberculosis. Chest. 2000;118(5):1355-64.

51. Nagesh BS, Sehgal S, Jindal SK, Arora SK. Evaluation of polymerase chain reaction for detection of Mycobacterium tuberculosis in pleural fluid. Chest. 2001;119(6):1737-41.

52. Reechaipichitkul W, Kawamatawong T, Teerajetgul Y, Patjanasoontorn B. Diagnostic role of pleural fluid

adenosine deaminase in tuberculous pleural effusion. Southeast Asian J Trop Med Public Health. 2001;32(2):383-9.

53. Sharma SK, Suresh V, Mohan A, Kaur P, Saha P, Kumar A, et al. A prospective study of sensitivity and specificity of adenosine deaminase estimation in the diagnosis of tuberculosis pleural effusion. Indian J Chest Dis Allied Sci. 2001;43(3):149-55.
54. Yamada Y, Nakamura A, Hosoda M, Kato T, Asano T, Tonegawa K, et al. Cytokines in pleural liquid for diagnosis of tuberculous pleurisy. Respir Med. 2001;95(7):577-81.
55. Andreasyan NA, Hairapetian HL, Sargisova YG, Mardanyan SS, Badalyan LT, Khanoyan AS. Activity of adenosine deaminase and its isoforms in pleural fluid in tuberculous pleuritis. Med Sci Monit. 2002;8(10):Cr708-12.
56. Jimenez D, Perez-Rodriguez E, Diaz G, Fogue L, Light RW. Determining the optimal number of specimens to obtain with needle biopsy of the pleura. Respir Med. 2002;96(1):14-7.
57. Diacon AH, Van de Wal BW, Wyser C, Smedema JP, Bezuidenhout J, Bolliger CT, et al. Diagnostic tools in tuberculous pleurisy: a direct comparative study. Eur Respir J. 2003;22(4):589-91.
58. Lima DM, Colares JK, da Fonseca BA. Combined use of the polymerase chain reaction and detection of adenosine deaminase activity on pleural fluid improves the rate of diagnosis of pleural tuberculosis. Chest. 2003;124(3):909-14.
59. Porcel JM, Vives M. Differentiating tuberculous from malignant pleural effusions: a scoring model. Med Sci Monit. 2003;9(5):Cr175-80.
60. Tahhan M, Ugurman F, Gozu A, Akkalyoncu B, Samurkasoglu B. Tumour necrosis factor-alpha in comparison to adenosine deaminase in tuberculous pleuritis. Respiration. 2003;70(3):270-4.
61. Chen ML, Yu WC, Lam CW, Au KM, Kong FY, Chan AY. Diagnostic value of pleural fluid adenosine deaminase activity in tuberculous pleurisy. Clin Chim Acta. 2004;341(1-2):101-7.
62. Ghanei M, Aslani J, Bahrami H, Adhami H. Simple method for rapid diagnosis of tuberculosis pleuritis: a statistical approach. Asian Cardiovasc Thorac Ann. 2004;12(1):23-9.
63. Poyraz B, Kaya A, Ciledag A, Oktem A, Gonullu U. Diagnostic significance of gamma-interferon in tuberculous pleurisy. Tuberk Toraks. 2004;52(3):211-7.
64. Gaga M, Papamichalis G, Bakakos P, Latsi P, Samara I, Koulouris NG, et al. Tuberculous effusion: ADA activity correlates with CD4+ cell numbers in the fluid and the pleura. Respiration. 2005;72(2):160-5.
65. Gao ZC, Tian RX. Clinical investigation on diagnostic value of interferon-gamma, interleukin-12 and adenosine deaminase isoenzyme for tuberculous pleurisy. Chin Med J (Engl). 2005;118(3):234-7.
66. Moon JW, Chang YS, Kim SK, Kim YS, Lee HM, Kim SK, et al. The clinical utility of polymerase chain reaction for the diagnosis of pleural tuberculosis. Clin Infect Dis. 2005;41(5):660-6.
67. Okamoto M, Kawabe T, Iwasaki Y, Hara T, Hashimoto N, Imaizumi K, et al. Evaluation of interferon-gamma, interferon-gamma-inducing cytokines, and interferon-gamma-inducible chemokines in tuberculous pleural effusions. J Lab Clin Med. 2005;145(2):88-93.
68. Sharma SK, Banga A. Pleural fluid interferon-gamma and adenosine deaminase levels in tuberculosis pleural effusion: a cost-effectiveness analysis. J Clin Lab Anal. 2005;19(2):40-6.
69. Tozkoparan E, Deniz O, Cakir E, Yaman H, Ciftci F, Gumus S, et al. The diagnostic values of serum, pleural fluid and urine neopterin measurements in tuberculous pleurisy. Int J Tuberc Lung Dis. 2005;9(9):1040-5.
70. Celik G, Kaya A, Poyraz B, Ciledag A, Elhan AH, Oktem A, et al. Diagnostic value of leptin in tuberculous pleural effusions. Int J Clin Pract. 2006;60(11):1437-42.
71. Mishra OP, Kumar R, Ali Z, Prasad R, Nath G. Evaluation of polymerase chain reaction and adenosine deaminase assay for the diagnosis of tuberculous effusions in children. Arch Dis Child. 2006;91(12):985-9.
72. Morimoto T, Takanashi S, Hasegawa Y, Fujimoto K, Okudera K, Hayashi A, et al. Level of antibodies against mycobacterial glycolipid in the effusion for diagnosis of tuberculous pleural effusion. Respir Med. 2006;100(10):1775-80.
73. Antonangelo L, Vargas FS, Seiscento M, Bombarda S, Teixera L, Sales RK. Clinical and laboratory parameters in the differential diagnosis of pleural effusion secondary to tuberculosis or cancer. Clinics (Sao Paulo). 2007;62(5):585-90.
74. Ariga H, Kawabe Y, Nagai H, Kurashima A, Masuda K, Matsui H, et al. Diagnosis of active tuberculous serositis by antigen-specific interferon-gamma response of cavity fluid cells. Clin Infect Dis. 2007;45(12):1559-67.
75. Cok G, Parildar Z, Basol G, Kabaroglu C, Bayindir U, Habif S, et al. Pleural fluid neopterin levels in tuberculous pleurisy. Clin Biochem. 2007;40(12):876-80.
76. Daniil ZD, Zintzaras E, Kiropoulos T, Papaioannou AI, Koutsokera A, Kastanis A, et al. Discrimination of exudative pleural effusions based on multiple biological parameters. Eur Respir J. 2007;30(5):957-64.
77. Lamsal M, Gautam N, Bhatta N, Majhi S, Baral N, Bhattacharya SK. Diagnostic utility of adenosine deaminase (ADA) activity in pleural fluid and serum of tuberculous and non-tuberculous respiratory disease

patients. Southeast Asian J Trop Med Public Health. 2007;38(2):363-9.
78. Moon JW. Sensitivity of polymerase chain reaction for pleural tuberculosis according to the amount of pleural effusion specimens. Tuberc Respir Dis. 2007;62(3):184-91.
79. Neves DD, Dias RM, Cunha AJ. Predictive model for the diagnosis of tuberculous pleural effusion. Braz J Infect Dis. 2007;11(1):83-8.
80. Trajman A, Kaisermann C, Luiz RR, Sperhacke RD, Rossetti ML, Feres Saad MH, et al. Pleural fluid ADA, IgA-ELISA and PCR sensitivities for the diagnosis of pleural tuberculosis. Scand J Clin Lab Invest. 2007;67(8):877-84.
81. Xue K, Xiong S, Xiong W. Clinical value of vascular endothelial growth factor combined with interferon-gamma in diagnosing malignant pleural effusion and tuberculous pleural effusion. J Huazhong Univ Sci Technolog Med Sci. 2007;27(5):495-7.
82. Baba K, Hoosen AA, Langeland N, Dyrhol-Riise AM. Adenosine deaminase activity is a sensitive marker for the diagnosis of tuberculous pleuritis in patients with very low CD4 counts. PLoS One. 2008;3(7):e2788.
83. Bandyopadhyay D, Gupta S, Banerjee S, Gupta S, Ray D, Bhattacharya S, et al. Adenosine deaminase estimation and multiplex polymerase chain reaction in diagnosis of extra-pulmonary tuberculosis. Int J Tuberc Lung Dis. 2008;12(10):1203-8.
84. Krenke R, Safianowska A, Paplinska M, Nasilowski J, Dmowska-Sobstyl B, Bogacka-Zatorska E, et al. Pleural fluid adenosine deaminase and interferon gamma as diagnostic tools in tuberculosis pleurisy. J Physiol Pharmacol. 2008;59 Suppl 6:349-60.
85. Verma SK, Dubey AL, Singh PA, Tewerson SL, Sharma D. Adenosine deaminase (ADA) level in tubercular pleural effusion. Lung India. 2008;25(3):109-10.
86. Zaric B, Kuruc V, Milovancev A, Markovic M, Sarcev T, Canak V, et al. Differential diagnosis of tuberculous and malignant pleural effusions: what is the role of adenosine deaminase? Lung. 2008;186(4):233-40.
87. Chang HK, Eun KM, Sung HP, Yong IH, Seung HJ, Yong BP, et al. Usefulness of the pleural fluid adenosine deaminase with lymphocyte/neutrophil ratio in the diagnosis of tuberculous pleurisy for a region of intermediate prevalence of tuberculosis. Tuberc Respir Dis. 2009;66(6):437-43.
88. Dheda K, Van-Zyl Smit RN, Sechi LA, Badri M, Meldau R, Symons G, et al. Clinical diagnostic utility of IP-10 and LAM antigen levels for the diagnosis of tuberculous pleural effusions in a high burden setting. PLoS One. 2009;4(3):e4689.
89. Kupeli E, Karnak D, Elgun S, Arguder E, Kayacan O. Concurrent measurement of adenosine deaminase and dipeptidyl peptidase IV activity in the diagnosis of tuberculous pleural effusion. Diagn Microbiol Infect Dis. 2009;65(4):365-71.
90. Valdes L, San Jose E, Alvarez Dobano JM, Golpe A, Valle JM, Penela P, et al. Diagnostic value of interleukin-12 p40 in tuberculous pleural effusions. Eur Respir J. 2009;33(4):816-20.
91. Zemlin AE, Burgess LJ, Carstens ME. The diagnostic utility of adenosine deaminase isoenzymes in tuberculous pleural effusions. Int J Tuberc Lung Dis. 2009;13(2):214-20.
92. Ciledag A, Kaya A, Erol S, Sen E, Celik G, Cesur S, et al. The comparison of pleural fluid TNF-alpha and IL-10 levels with ADA in tuberculous pleural effusion. Curr Med Chem. 2010;17(19):2096-100.
93. Gupta BK, Bharat V, Bandyopadhyay D. Role of Adenosine Deaminase Estimation in Differentiation of Tuberculous and Non-tuberculous Exudative Pleural Effusions. J Clin Med Res. 2010;2(2):79-84.
94. Katiyar SK, Sampath A, Bihari S, Mamtani M, Kulkarni H. Using a whole-blood interferon-gamma assay to improve diagnosis of tuberculous pleural effusion. Eur Respir J. 2010;36(3):679-81.
95. Pandit S, Chaudhuri AD, Datta SB, Dey A, Bhanja P. Role of pleural biopsy in etiological diagnosis of pleural effusion. Lung India. 2010;27(4):202-4.
96. Porcel JM, Esquerda A, Bielsa S. Diagnostic performance of adenosine deaminase activity in pleural fluid: a single-center experience with over 2100 consecutive patients. Eur J Intern Med. 2010;21(5):419-23.
97. Song D, Lun AR, Chiu W. Diazyme adenosine deaminase in the diagnosis of tuberculous pleural effusion: Method evaluation and clinical experiences in a New Zealand population. N Z J Med Lab Sci. 2010;64(1):11-3.
98. Valdes L, San Jose ME, Pose A, Gude F, Gonzalez-Barcala FJ, Alvarez-Dobano JM, et al. Diagnosing tuberculous pleural effusion using clinical data and pleural fluid analysis A study of patients less than 40 years-old in an area with a high incidence of tuberculosis. Respir Med. 2010;104(8):1211-7.
99. Wu SH, Li CT, Lin CH, Chu JJ, Cheng ML, Lin KH. Soluble Fas ligand is another good diagnostic marker for tuberculous pleurisy. Diagn Microbiol Infect Dis. 2010;68(4):395-400.
100. Ambade V, Arora MM, Rai SP, Nikumb SK, Basannar DR. Markers for differentiation of tubercular pleural effusion from non-tubercular effusion. Med J Armed Forces India. 2011;67(4):338-42.
101. Bhutia ND, Singh YT, Shyamsunder C, Satyakumar T, Singh MA, Keithellakpam S. Evaluation of adenosine deaminase (ADA) activity in the diagnosis of tuberculous pleural effusion in children. J Med Soc. 2011;25(1):48-51.
102. Kalantri Y, Hemvani N, Chitnis DS. Evaluation of real-time polymerase chain reaction, interferon-gamma,

adenosine deaminase, and immunoglobulin A for the efficient diagnosis of pleural tuberculosis. Int J Infect Dis. 2011;15(4):e226-31.

103. Liu YC, Shin-Jung Lee S, Chen YS, Tu HZ, Chen BC, Huang TS. Differential diagnosis of tuberculous and malignant pleurisy using pleural fluid adenosine deaminase and interferon gamma in Taiwan. J Microbiol Immunol Infect. 2011;44(2):88-94.

104. Ogata Y, Aoe K, Hiraki A, Murakami K, Kishino D, Chikamori K, et al. Is adenosine deaminase in pleural fluid a useful marker for differentiating tuberculosis from lung cancer or mesothelioma in Japan, a country with intermediate incidence of tuberculosis? Acta Med Okayama. 2011;65(4):259-63.

105. Yildiz PB, Yazar EE, Gorgun D, Secik F, Cakir G. Predictive role of adenosine deaminase for differential diagnosis of tuberculosis and malignant pleural effusion in Turkey. Asian Pac J Cancer Prev. 2011;12(2):419-23.

106. Antonangelo L, Vargas FS, Genofre EH, Oliveira CM, Teixeira LR, Sales RK. Differentiating between tuberculosis-related and lymphoma-related lymphocytic pleural effusions by measuring clinical and laboratory variables: is it possible? J Bras Pneumol. 2012;38(2):181-7.

107. Cirak AK, Komurcuoglu B, Tekgul S, Bilaceroglu S, Tasdogen N, Gunduz A. The diagnostic efficiency of QuantiFERONTB-Gold test in the diagnosis of tuberculous pleurisy. Int J Mycobacteriol. 2012;1(4):180-4.

108. Demirer E, Miller AC, Kunter E, Kartaloglu Z, Barnett SD, Elamin EM. Predictive models for tuberculous pleural effusions in a high tuberculosis prevalence region. Lung. 2012;190(2):239-48.

109. Devkota KC, Shyam BK, Sherpa K, Ghimire P, Sherpa MT, Shrestha R, et al. Significance of adenosine deaminase in diagnosing tuberculous pleural effusion. Nepal Med Coll J. 2012;14(2):149-52.

110. Garcia-Zamalloa A, Taboada-Gomez J. Diagnostic accuracy of adenosine deaminase and lymphocyte proportion in pleural fluid for tuberculous pleurisy in different prevalence scenarios. PLoS One. 2012;7(6):e38729.

111. Kashiwabara K, Okamoto T, Yamane H. When pleural potassium exceeds 5.0 mEq/L, high pleural adenosine deaminase levels do not necessarily indicate tuberculous pleuritis. Respirology. 2012;17(1):92-8.

112. Kumar M, Sharma R, Dubey M, Banu N, Sharma S. Mycobacterium tuberculosis detection in pleural effusion by adenosine deaminase assay. Asian J Microbiol Biotechnol Environ Sci. 2012;14(2):181-6.

113. Pal S, Gupta S. Adenosine deaminase - the non-invasive marker of tuberculosis. J Indian Med Assoc. 2012;110(1):16-8.

114. Wang H, Yue J, Yang J, Gao R, Liu J. Clinical diagnostic utility of adenosine deaminase, interferon-gamma, interferon-gamma-induced protein of 10 kDa, and dipeptidyl peptidase 4 levels in tuberculous pleural effusions. Heart Lung. 2012;41(1):70-5.

115. Kelam MA, Ganie FA, Shah BA, Ganie SA, Wani ML, Wani NU, et al. The diagnostic efficacy of adenosine deaminase in tubercular effusion. Oman Med J. 2013;28(6):417-21.

116. Keng LT, Shu CC, Chen JY, Liang SK, Lin CK, Chang LY, et al. Evaluating pleural ADA, ADA2, IFN-gamma and IGRA for diagnosing tuberculous pleurisy. J Infect. 2013;67(4):294-302.

117. Khan FY, Hamza M, Omran AH, Saleh M, Lingawi M, Alnaqdy A, et al. Diagnostic value of pleural fluid interferon-gamma and adenosine deaminase in patients with pleural tuberculosis in Qatar. Int J Gen Med. 2013;6:13-8.

118. Khow-Ean N, Booraphun S, Aekphachaisawat N, Sawanyawisuth K. Adenosine deaminase activity level as a tool for diagnosing tuberculous pleural effusion. Southeast Asian J Trop Med Public Health. 2013;44(4):655-9.

119. Lee KS, Kim HR, Kwak S, Choi KH, Cho JH, Lee YJ, et al. Association between elevated pleural interleukin-33 levels and tuberculous pleurisy. Ann Lab Med. 2013;33(1):45-51.

120. Sahn SA, Huggins JT, San Jose ME, Alvarez-Dobano JM, Valdes L. Can tuberculous pleural effusions be diagnosed by pleural fluid analysis alone? Int J Tuberc Lung Dis. 2013;17(6):787-93.

121. Tay TR, Tee A. Factors affecting pleural fluid adenosine deaminase level and the implication on the diagnosis of tuberculous pleural effusion: a retrospective cohort study. BMC Infect Dis. 2013;13:546.

122. Wu YB, Ye ZJ, Qin SM, Wu C, Chen YQ, Shi HZ. Combined detections of interleukin 27, interferon-gamma, and adenosine deaminase in pleural effusion for diagnosis of tuberculous pleurisy. Chin Med J (Engl). 2013;126(17):3215-21.

123. Abrao FC, de Abreu IR, Miyake DH, Busico MA, Younes RN. Role of adenosine deaminase and the influence of age on the diagnosis of pleural tuberculosis. Int J Tuberc Lung Dis. 2014;18(11):1363-9.

124. Anwar MU, Sarah, Afzal A, Kamal MU. Adenosine deaminase as a diagnostic aid to ascertain tuberculous etiology of pleural effusions. Pak J Med Health Sci. 2014;8(3):702-5.

125. Kong XL, Zeng HH, Chen Y, Liu TT, Shi ZH, Zheng DY, et al. The visual diagnosis of tuberculous pleuritis under medical thoracoscopy: a retrospective series of 91 cases. Eur Rev Med Pharmacol Sci. 2014;18(10):1487-95.

126. Li M, Wang H, Wang X, Huang J, Wang J, Xi X. Diagnostic accuracy of tumor necrosis factor-alpha, interferon-gamma, interleukin-10 and adenosine deaminase 2 in differential diagnosis between tuberculous

pleural effusion and malignant pleural effusion. J Cardiothorac Surg. 2014;9:118.
127. Liao M, Yang Q, Zhang J, Zhang M, Deng Q, Liu H, et al. Gamma interferon immunospot assay of pleural effusion mononuclear cells for diagnosis of tuberculous pleurisy. Clin Vaccine Immunol. 2014;21(3):347-53.
128. Mehta AA, Gupta AS, Ahmed S, Rajesh V. Diagnostic utility of adenosine deaminase in exudative pleural effusions. Lung India. 2014;31(2):142-4.
129. Meldau R, Peter J, Theron G, Calligaro G, Allwood B, Symons G, et al. Comparison of same day diagnostic tools including Gene Xpert and unstimulated IFN-gamma for the evaluation of pleural tuberculosis: a prospective cohort study. BMC Pulm Med. 2014;14:58.
130. Rahim A, Ahmad A, Qureshi MA. Level of serum adenosine deaminase in cases of tuberculous pleural effusion. Pak J Med Health Sci. 2014;8(4):994-6.
131. Reis R, Costa AS, Conde B. Pleural adenosine deaminase in the diagnostic workup of tuberculous pleural effusion. Rev Port Pneumol. 2014;20(4):228-9.
132. Sanchez-Otero N, Rodriguez-Berrocal FJ, de la Cadena MP, Botana-Rial MI, Cordero OJ. Evaluation of pleural effusion sCD26 and DPP-IV as diagnostic biomarkers in lung disease. Sci Rep. 2014;4:3999.
133. Sethi S, Kaur J, Yadav R, Dhatwalia SK, Mewara A, Rana S, et al. Combination of adenosine-deaminase and nucleic acid amplification assays for diagnosing tuberculous pleural effusion. J Infect. 2014;69(1):99-101.
134. Trajman A, da Silva Santos Kleiz de Oliveira EF, Bastos ML, Belo Neto E, Silva EM, da Silva Lourenco MC, et al. Accuracy of polimerase chain reaction for the diagnosis of pleural tuberculosis. Respir Med. 2014;108(6):918-23.
135. Valdes L, San Jose E, Ferreiro L, Golpe A, Gude F, Alvarez-Dobano JM, et al. Interleukin 27 could be useful in the diagnosis of tuberculous pleural effusions. Respir Care. 2014;59(3):399-405.
136. Yurt S, Kucukergin C, Yigitbas BA, Seckin S, Tigin HC, Kosar AF. Diagnostic utility of serum and pleural levels of adenosine deaminase 1-2, and interferon-gamma in the diagnosis of pleural tuberculosis. Multidiscip Respir Med. 2014;9(1):12.
137. Agha MA, El-Habashy MM, Helwa MA, Habib RM. Role of thoracentesis in the management of tuberculous pleural effusion. Egypt J Chest Dis and Tuberc. 2015;64(1):97-102.
138. Ali AHK, Mahmoud TM, Ahmed H. Differential diagnostic efficiency of T cells subsets versus interferon-gamma, tumor necrosis factor-alpha and adenosine deaminase in distinguishing tuberculous from malignant pleural effusions. Egypt J Chest Dis and Tuberc. 2015;64(3):645-51.
139. Arnold DT, Bhatnagar R, Fairbanks LD, Zahan-Evans N, Clive AO, Morley AJ, et al. Pleural fluid adenosine deaminase (pfADA) in the diagnosis of tuberculous effusions in a low incidence population. PLoS One. 2015;10(2):e0113047.
140. Behrsin RF, Junior CT, Cardoso GP, Barillo JL, de Souza JB, de Araujo EG. Combined evaluation of adenosine deaminase level and histopathological findings from pleural biopsy with Cope's needle for the diagnosis of tuberculous pleurisy. Int J Clin Exp Pathol. 2015;8(6):7239-46.
141. Farhana A, Ghosh CK, Rehena Z, Ferdousi S, Alam MB, Mahmuduzzaman M, et al. Comparative study of adenosine deaminase and other conventional diagnostic parameters in diagnosis of tuberculous pleural effusion. Mymensingh Med J. 2015;24(3):550-7.
142. He Y, Zhang W, Huang T, Wang X, Wang M. Evaluation of a diagnostic flow chart applying medical thoracoscopy, adenosine deaminase and T-SPOT.TB in diagnosis of tuberculous pleural effusion. Eur Rev Med Pharmacol Sci. 2015;19(19):3563-8.
143. Klimiuk J, Krenke R, Safianowska A, Korczynski P, Chazan R. Diagnostic performance of different pleural fluid biomarkers in tuberculous pleurisy. Adv Exp Med Biol. 2015;852:21-30.
144. Kosar F, Yurt S, Arpinar Yigitbas B, Seker B, Kutbay Ozcelik H, Uzun H. The comparative value of pleural fluid adenosine deaminase and neopterin levels in diagnostic utility of pleural tuberculosis. Tuberk Toraks. 2015;63(4):243-9.
145. Kumar S, Agarwal R, Bal A, Sharma K, Singh N, Aggarwal AN, et al. Utility of adenosine deaminase (ADA), PCR and thoracoscopy in differentiating tuberculous and non-tuberculous pleural effusion complicating chronic kidney disease. Indian J Med Res. 2015;141(3):308-14.
146. Li D, Shen Y, Fu X, Li M, Wang T, Wen F. Combined detections of interleukin-33 and adenosine deaminase for diagnosis of tuberculous pleural effusion. Int J Clin Exp Pathol. 2015;8(1):888-93.
147. Saiphoklang N, Kanitsap A, Nambunchu A. Differences in clinical manifestations and pleural fluid charateristics between tuberculous and malignant pleural effusions. Southeast Asian J Trop Med Public Health. 2015;46(3):496-503.
148. Shu CC, Wang JY, Hsu CL, Keng LT, Tsui K, Lin JF, et al. Diagnostic role of inflammatory and anti-inflammatory cytokines and effector molecules of cytotoxic T lymphocytes in tuberculous pleural effusion. Respirology. 2015;20(1):147-54.
149. Skouras VS, Magkouta SF, Psallidas I, Tsilioni I, Maragozidis P, Gourgoulianis KI, et al. Interleukin-27 improves the ability of adenosine deaminase to rule out tuberculous pleural effusion regardless of pleural

tuberculosis prevalence. Infect Dis (Lond). 2015;47(7):477-83.

150. Tural Onur S, Sokucu SN, Dalar L, Seyhan EC, Akbas A, Altin S. Are soluble IL-2 receptor and IL-12p40 levels useful markers for diagnosis of tuberculous pleurisy? Infect Dis (Lond). 2015;47(3):150-5.
151. Yoshino Y, Wakabayashi Y, Seo K, Koga I, Kitazawa T, Ota Y. Hyaluronic Acid concentration in pleural fluid: diagnostic aid for tuberculous pleurisy. J Clin Med Res. 2015;7(1):41-4.
152. Biswas B, Sharma SK, Negi RS, Gupta N, Jaswal VM, Niranjan N. Pleural effusion: Role of pleural fluid cytology, adenosine deaminase level, and pleural biopsy in diagnosis. J Cytol. 2016;33(3):159-62.
153. Corral-Gudino L, Garcia-Zamalloa A, Prada-Gonzalez C, Bielsa S, Alexis D, Taboada-Gomez J, et al. Development and validation of the COMPLES score for differentiating between tuberculous effusions with low pleural pH or glucose and complicated parapneumonic effusions. Lung. 2016;194(5):847-54.
154. Kim MC, Kim SM, Lee SO, Choi SH, Kim YS, Woo JH, et al. A diagnostic algorithm for tuberculous pleurisy using the ELISPOT assay on peripheral blood and pleural effusion. Infect Dis (Lond). 2016;48(9):688-94.
155. Lee J, Lim JK, Lee SY, Yoo SS, Lee SY, Cha SI, et al. Neutrophilic loculated tuberculous pleural effusion: incidence, characteristics and differentiation from complicated parapneumonic effusion. Am J Med Sci. 2016;351(2):153-9.
156. Liu Y, Ou Q, Zheng J, Shen L, Zhang B, Weng X, et al. A combination of the QuantiFERON-TB Gold In-Tube assay and the detection of adenosine deaminase improves the diagnosis of tuberculous pleural effusion. Emerg Microbes Infect. 2016;5(8):e83.
157. Mallik M, Bhartiya R, Singh R, Kumar M, Bariar NK. Adenosine deaminase: a sensitive and cost-effective method for the detection of tuberculous pleural effusion in a developing state like Bihar, India. Ann Trop Med Public Health. 2016;9(3):170-3.
158. Michot JM, Madec Y, Bulifon S, Thorette-Tcherniak C, Fortineau N, Noel N, et al. Adenosine deaminase is a useful biomarker to diagnose pleural tuberculosis in low to medium prevalence settings. Diagn Microbiol Infect Dis. 2016;84(3):215-20.
159. Rahman MF, Anand P, Arya ML. Study of adenosine deaminase activity in tuberculour pleural effusion and other respiratory diseases. Indian J Public Health Res Dev. 2016;7(2):85-9.
160. Saiphoklang N, Kanitsap A, Ruchiwit P. Diagnostic value of pleural fluid adenosine deaminase in tuberculous pleuritis at Thammasat University Hospital. J Med Assoc Thai. 2016;99(Suppl 4):S1-S9.
161. Suleman A, Kamal M, Abbasi MA. Diagnostic utility of pleural fluid adenosine deaminase level In tuberculous pleural effusion. J Ayub Med Coll Abbottabad. 2016;28(2):245-8.
162. Che N, Yang X, Liu Z, Li K, Chen X. Rapid detection of cell-free Mycobacterium tuberculosis DNA in tuberculous pleural effusion. J Clin Microbiol. 2017;55(5):1526-32.
163. Chung W, Jung Y, Lee K, Park J, Sheen S, Park K. CXCR3 ligands in pleural fluid as markers for the diagnosis of tuberculous pleural effusion. Int J Tuberc Lung Dis. 2017;21(12):1300-6.
164. El Hoshy MS, Abdallah AA, Abd Elhamid SM. Comparison of the diagnostic utility of ADA and CA125 in tuberculous effusion. Egypt J Chest Dis Tuberc. 2017;66(2):299-305.
165. Kim CH, Park JE, Yoo SS, Lee SY, Cha SI, Park JY, et al. Usefulness of serum lactate dehydrogenase/pleural fluid adenosine deaminase ratio for differentiating Mycoplasma pneumoniae parapneumonic effusion and tuberculous pleural effusion. J Infect. 2017;75(6):581-3.
166. Sivakumar P, Marples L, Breen R, Ahmed L. The diagnostic utility of pleural fluid adenosine deaminase for tuberculosis in a low prevalence area. Int J Tuberc Lung Dis. 2017;21(6):697-701.
167. Xu HY, Li CY, Su SS, Yang L, Ye M, Ye JR, et al. Diagnosis of tuberculous pleurisy with combination of adenosine deaminase and interferon-gamma immunospot assay in a tuberculosis-endemic population: A prospective cohort study. Medicine (Baltimore). 2017;96(47):e8412.
168. Zhang Q, Zhou C. Comparison of laboratory testing methods for the diagnosis of tuberculous pleurisy in China. Sci Rep. 2017;7(1):4549.
169. Blakiston M, Chiu W, Wong C, Morpeth S, Taylor S. Diagnostic performance of pleural fluid adenosine deaminase for tuberculous pleural effusion in a low-incidence setting. J Clin Microbiol. 2018;56(8).
170. Chang KC, Chan MC, Leung WM, Kong FY, Mak CM, Chen SP, et al. Optimising the utility of pleural fluid adenosine deaminase for the diagnosis of adult tuberculous pleural effusion in Hong Kong. Hong Kong Med J. 2018;24(1):38-47.
171. He J, Zhang R, Shen Y, Wan C, Zeng N, Qin J, et al. Diagnostic accuracy of interleukin-22 and adenosine deaminase for tuberculous pleural effusions. Curr Res Transl Med. 2018.
172. Hong JY, Park SY, Kim Y, Lee CY, Lee MG. Calpain and spectrin breakdown products as potential biomarkers in tuberculous pleural effusion. J Thorac Dis. 2018;10(5):2558-66.
173. Santos AP, Correa RDS, Ribeiro-Alves M, Soares da Silva ACO, Mafort TT, Leung J, et al. Application of Venn's diagram in the diagnosis of pleural tuberculosis using IFN-gamma, IP-10 and adenosine deaminase. PLoS One. 2018;13(8):e0202481.
174. Wang W, Zhou Q, Zhai K, Wang Y, Liu JY, Wang XJ, et al. Diagnostic accuracy of interleukin 27 for tuberculous pleural effusion: two prospective studies and one meta-analysis. Thorax. 2018;73(3):240-7.
