# Supplementary material for: Adenosine deaminase for diagnosis of tuberculous pleural effusion: A systematic review and meta-analysis
Source: PLoS One. 2019 Mar 26;14(3):e0213728. doi: 10.1371/journal.pone.0213728 (PMC6435228; doi:10.1371/journal.pone.0213728)
Supplement: S2 Table — (PDF) [file pone.0213728.s006.pdf]

S2 Table. Characteristics of studies included in data synthesis.

Author, year	Country of study	Prospective study	Case control design	Inclusion criteria	Exclusion criteria	HIV+ patients	Standard for TB diagnosis	Definite TB*	Funding source
Piras, 1978[1]	Italy	Yes	No	NS	NS	NS	NS	NS	NS
Blake, 1982[2]	South Africa	Yes	No	NS	NS	NS	CMP	NS	NS
Maritz, 1982[3]	South Africa	Yes	No	NS	Incomplete data, no final diagnosis	NS	CMP	68.2%	NS
Petterson, 1984[4]	Finland	Yes	No	Inpatients with effusion	NS	NS	CMP	73.7%	Multiple professional agencies
Niwa, 1985[5]	Japan	No	Yes	Confirmed tuberculous or malignant effusion	NS	NS	MP	100%	NS
Raj, 1985[6]	India	No	No	NS	NS	NS	MP	100%	NS
Sinha, 1987[7]	India	Yes	No	Patients with first episode of pleural effusion	No final diagnosis	NS	CMP	NS	NS
Strankinga, 1987[8]	Netherlands	Yes	No	Inpatients with effusion	Hematologic malignancy, rheumatoid arthritis (due to very few patients)	NS	MP	100%	NS
Teo, 1987[9]	Singapore	Yes	No	Patients >12 years admitted with effusion	NS	NS	CMP	88.0%	Government
van Keimpema, 1987[10]	Netherlands	No	No	Stored pleural fluid samples	NS	NS	M	100%	NS
Fontan Bueso, 1988[11]	Spain	Yes	No	Inpatients with effusion	NS	NS	MP	100%	NS
Kao, 1988[12]	Taiwan	Yes	No	Inpatients with effusion	NS	NS	MP	100%	NS
Kim, 1988[13]	Korea	Yes	No	Inpatients with effusion	NS	NS	NS	NS	NS
Tamura, 1988[14]	Japan	No	No	NS	NS	NS	CMP	NS	NS
Gilhotra, 1989[15]	India	No	No	Pleural effusion with proven histo/cytological diagnosis	NS	NS	P	100%	NS
Hsu, 1989[16]	Taiwan	No	No	Inpatients with effusion	NS	NS	MP	100%	NS
Moriwaki, 1989[17]	Japan	No	Yes	NS	NS	NS	MP	100%	NS

Segura, 1989[18]	Spain	Yes	No	Inpatients with effusion	NS	NS	MP	100%	NS
Gourgoulianis, 1990[19]	Greece	NS	NS	NS	NS	NS	MP	100%	NS
Gupta, 1990[20]	India	Yes	No	NS	NS	NS	MP	100%	NS
Banales, 1991[21]	Canada	Yes	No	Inpatients with exudative effusion	NS	NS	MP	100%	Government (foreign)
Lopez Jimenez, 1991[22]	Spain	Yes	No	NS	NS	NS	NS	NS	NS
Maartens, 1991[23]	South Africa	Yes	No	Adult patients undergoing pleural aspiration/biopsy, patients with malignancy having new effusion	Empyema fluid judged unsuitable for ADA estimation	NS	MP	100%	NS
Hara, 1992[24]	Japan	Yes	No	NS	NS	NS	MP	100%	NS
Kaur, 1992[25]	India	Yes	No	Inpatients with effusion	NS	NS	CMP	65.6%	Institutional, Government
Muranishi, 1992[26]	Japan	Yes	No	Inpatients with effusion	NS	NS	CMP	52.9%	NS
Nagaraja, 1992[27]	India	Yes	No	NS	Empyema, coexistent TB and malignancy	NS	CMP	66.7%	NS
Perez-Rodriguez, 1992[28]	Spain	Yes	No	NS	NS	NS	NS	NS	NS
Prasad, 1992[29]	India	Yes	No	Inpatients with effusion	NS	NS	MP	100%	NS
Hsu, 1993[30]	Taiwan	Yes	Yes	Inpatients with effusion	NS	NS	MP	100%	NS
Valdes, 1993[31]	Spain	Yes	No	Inpatients with effusion	No definite diagnosis	NS	MP	100%	NS
Aoki, 1994[32]	Japan	Yes	No	NS	NS	NS	MP	100%	Corporate
Chiang, 1994[33]	Taiwan	No	No	NS	NS	NS	MP	100%	NS
De Olivera, 1994[34]	Brazil	Yes	No	Patients undergoing pleural aspiration	Cardiac failure, small effusion	NS	CMP	48.1%	NS
Richter, 1994[35]	Tanzania	Yes	No	Inpatients with effusion	Cardiac failure, patients on anti-tubercular therapy	49/75	CMP	75.0%	Professional association
Burgess, 1995[36]	South Africa	Yes	No	Patients undergoing pleural aspiration	NS	NS	CMP	76.6%	NS
Querol, 1995[37]	Spain	Yes	No	NS	NS	NS	MP	100%	NS
Valdes, 1995[38]	Spain	Yes	No	Inpatients <=35 years with effusion	Empyema	NS	MP	100%	NS

Orphanidou, 1996[39]	Greece	NS	NS	NS	NS	NS	MP	100%	NS
Valdes, 1996[40]	Spain	Yes	No	Inpatients with effusion	No definite diagnosis	None	MP	100%	NS
Villena, 1996[41]	Spain	Yes	No	NS	Empyema	7/49	MP	100%	Government
Ogawa, 1997[42]	Japan	No	No	NS	No definite diagnosis	NS	MP	100%	NS
Kuralay, 1998[43]	Turkey	NS	No	Inpatients with effusion	NS	NS	MP	100%	NS
Ghelani, 1999[44]	India	Yes	No	NS	Past history of tuberculosis	13/81	CMP	63.0%	NS
Perez-Rodriguez, 1999[45]	Spain	Yes	No	NS	NS	NS	CMP	96.2%	NS
Riantawan, 1999[46]	Thailand	Yes	No	Inpatients with effusion	NS	37/89	MP	100%	NS
San Jose, 1999[47]	Spain	Yes	No	Inpatients with effusion	NS	NS	CMP	NS	Government
Gorguner, 2000[48]	Turkey	Yes	No	NS	No definite diagnosis	NS	MP	100%	NS
Prandaman, 2000[49]	India	Yes	No	NS	NS	NS	CMP	NS	Institutional
Villegas, 2000[50]	Columbia	Yes	No	Age >18 years	NS	NS	CMP	68.9%	NS
Nagesh, 2001[51]	India	Yes	No	Outpatients with effusion	Known definite etiology for effusion, patients on anti-tubercular therapy	NS	CMP	60.0%	NS
Reechaipichitkul, 2001[52]	Thailand	Yes	No	Exudative lymphocytic effusion	Bleeding diathesis	NS	CMP	70.0%	Institutional
Sharma, 2001[53]	India	Yes	No	Outpatients with effusion	NS	NS	CMP	21.3%	NS
Yamada, 2001[54]	Japan	No	No	NS	NS	NS	MP	100%	NS
Andreasyan, 2002[55]	Armenia	Yes	No	NS	NS	NS	CMP	NS	NS
Jimenez, 2002[56]	Spain	Yes	No	NS	NS	NS	MP	100%	NS
Diacon, 2003[57]	South Africa	Yes	No	Exudative pleural effusion	Immunocompromised or HIV seropositive patients	None	MP	100%	NS
Lima, 2003[58]	Brazil	Yes	No	Suspected tuberculous effusion	Symptoms for <4 weeks	NS	CMP	62.5%	NS
Porcel, 2003[59]	Spain	No	Yes	Tuberculous or malignant effusion	NS	NS	CMP	45.3%	NS
Tahhan, 2003[60]	Turkey	Yes	No	Exudative pleural effusion	No definite diagnosis	NS	MP	100%	NS

Chen, 2004[61]	Hong Kong	No	No	Inpatients with effusion	Non-Chinese patients, no definite diagnosis, hemothorax, transudates	NS	CMP	NS	NS
Ghanei, 2004[62]	Iran	Yes	No	NS	NS	NS	MP	100%	NS
Poyraz, 2004[63]	Turkey	Yes	No	Inpatients with effusion	NS	NS	P	100%	NS
Gaga, 2005[64]	Greece	Yes	Yes	NS	NS	None	CMP	94.4%	NS
Gao, 2005[65]	China	Yes	No	NS	NS	NS	CMP	NS	NS
Moon, 2005[66]	Korea	Yes	No	Suspected tuberculous effusion in >18 years age	NS	NS	CMP	71.9%	Institutional
Okamoto, 2005[67]	Japan	No	Yes	Exudative lymphocytic effusion	Diagnosis other than TB or malignancy, or not definite	NS	MP	100%	NS
Sharma, 2005[68]	India	Yes	No	NS	Immunosuppressive therapy, pregnancy, major organ dysfunction	None	MP	100%	NS
Tozkoparan, 2005[69]	Turkey	NS	Yes	NS	NS	NS	MP	100%	NS
Celik, 2006[70]	Turkey	NS	No	Exudative pleural effusion	NS	NS	MP	100%	NS
Mishra, 2006[71]	India	Yes	No	Children <14 years	NS	NS	CMP	35.0%	NS
Morimoto, 2006[72]	Japan	NS	No	NS	NS	NS	CMP	NS	NS
Antonangelo, 2007[73]	Brazil	Yes	Yes	Outpatients with effusion due to TB or malignancy	NS	NS	CMP	37.4%	NS
Ariga, 2007[74]	Japan	Yes	No	Inpatients with effusion	No definite diagnosis	None	CMP	100%	Government
Cok, 2007[75]	Turkey	NS	Yes	Tuberculous or malignant pleural effusion	Diabetes, chronic renal failure, chronic liver disease	NS	CMP	75.0%	NS
Daniil, 2007[76]	Greece	Yes	No	NS	NS	NS	MP	100%	NS
Lamsal, 2007[77]	Nepal	Yes	No	NS	NS	NS	CMP	13.8%	Institutional
Moon, 2007[78]	Korea	Yes	No	Suspected tuberculous effusion in >18 years age	NS	NS	CMP	53.8%	Institutional
Neves, 2007[79]	Brazil	Yes	No	NS	No definite diagnosis	NS	MP	100%	NS
Trajman, 2007[80]	Brazil	No	No	Inpatients with effusion	Anti-TB drugs for >7 days, no definite diagnosis	12/88	CMP	77.9%	Government

Xue, 2007[81]	China	Yes	Yes	Tuberculous or malignant pleural effusion	NS	NS	MP	100%	NS
Baba, 2008[82]	South Africa	No	No	Effusions for which pleural fluid ADA was estimated	NS	140/165	CMP	36.5%	NS
Bandyopadhyay, 2008[83]	India	Yes	Yes	Randomly selected patients with pleural effusion	NS	NS	CMP	14.7%	NS
Krenke, 2008[84]	Poland	Yes	No	Inpatients with effusion	No definite diagnosis	NS	MP	100%	NS
Verma, 2008[85]	India	Yes	No	NS	NS	NS	CMP	82.9%	NS
Zaric, 2008[86]	Serbia	Yes	Yes	Tuberculous or malignant effusion, >18 years age	Empyema, transudate, no definite diagnosis	NS	MP	100%	NS
Chang, 2009[87]	Korea	Yes	No	Inpatients with effusion	Transudates, pleural fluid leukocyte count not done	NS	CMP	79.6%	NS
Dheda, 2009[88]	South Africa	Yes	No	Suspected tuberculous effusion in >18 years age	Pregnant women, incomplete patient or sample data	20/51	CMP	87.3%	NS
Kupeli, 2009[89]	Turkey	Yes	No	Inpatients with effusion	No definite diagnosis	NS	CMP	38.9%	NS
Valdes, 2009[90]	Spain	Yes	No	Tuberculous, malignant or para-pneumonic effusion	NS	NS	MP	100%	NS
Zemlin, 2009[91]	South Africa	No	No	NS	Patients taking antitubercular treatment	NS	CMP	53.2%	NS
Ciledag, 2010[92]	Turkey	NS	No	NS	NS	NS	MP	100%	NS
Gupta, 2010[93]	India	Yes	No	Inpatients with effusion	NS	NS	CMP	16.1%	NS
Katiyar, 2010[94]	India	Yes	No	Exudative pleural effusion	Age <18 years, pregnancy, history of anti-TB therapy, on immunosuppressive therapy, HIV seropositive	None	CMP	73.1%	NS
Pandit, 2010[95]	India	Yes	No	NS	NS	NS	CMP	90.9%	NS
Porcel, 2010[96]	Spain	No	No	Pleural fluids for which ADA estimation was performed	NS	13/221	CMP	36.2%%	NS
Song, 2010[97]	New Zealand	No	No	Exudative pleural effusion	NS	NS	MP	100%	NS
Valdes, 2010[98]	Spain	Yes	No	Inpatients with effusion	NS	NS	MP	100%	NS
Wu, 2010[99]	Taiwan	No	No	NS	No definite diagnosis	NS	CMP	91.3%	Institutional

Ambade, 2011[100]	India	Yes	No	Inpatients with effusion	Subjects with incomplete data	NS	MP	100%	NS
Bhutia, 2011[101]	India	Yes	No	Outpatients with effusion	Age <13 years	NS	CMP	NS	NS
Kalantri, 2011[102]	India	Yes	No	NS	NS	NS	CMP	32.5%	Government
Liu, 2011[103]	Taiwan	Yes	No	Exudative and lymphocytic pleural effusion	NS	NS	MP	100%	Institutional
Ogata, 2011[104]	Japan	No	No	NS	NS	NS	MP	100%	Government
Yildiz, 2011[105]	Turkey	No	No	Tuberculous or malignant pleural effusion	Transudates, diagnosis other than TB/malignancy	NS	CMP	>94%	NS
Antonangelo, 2012[106]	Brazil	No	Yes	HIV seronegative patients with lymphocytic effusion due to tuberculosis or lymphoma	NS	None	CMP	NS	NS
Cirak, 2012[107]	Turkey	Yes	No	Inpatients with effusion	NS	NS	MP	100%	NS
Demirer, 2012[108]	Turkey	Yes	No	Effusion of indeterminate etiology in >18 years age	No definite diagnosis	NS	MP	100%	NS
Devkota, 2012[109]	Nepal	Yes	No	Inpatients with effusion	NS	NS	CMP	0.0%	NS
Garcia-Zamalloa, 2012[110]	Spain	No	No	NS	NS	NS	CMP	34.2%	NS
Kashiwabara, 2012[111]	Japan	No	No	Exudative pleural effusion	NS	NS	CMP	23.8%	NS
Kumar, 2012[112]	India	Yes	No	Exudative and lymphocytic pleural effusion	NS	NS	CMP	40.0%	NS
Pal, 2012[113]	India	No	No	NS	NS	NS	CMP	NS	NS
Wang, 2012[114]	China	Yes	No	NS	NS	NS	CMP	85.9%	NS
Kelam, 2013[115]	India	Yes	No	NS	NS	NS	CMP	82.1%	NS
Keng, 2013[116]	Taiwan	Yes	No	Exudative and lymphocytic pleural effusion	NS	1/88	CMP	83.9%	Government
Khan, 2013[117]	Qatar	Yes	No	Inpatients with effusion	NS	None	MP	100%	Professional association
Khow-Ean, 2013[118]	Thailand	No	No	Age >15 years undergoing pleural biopsy	NS	NS	P	100%	NS
Lee, 2013[119]	Korea	Yes	No	NS	No definite diagnosis	NS	MP	100%	Institutional
Sahn, 2013[120]	Spain	No	Yes	NS	No definite diagnosis	None	MP	100%	NS

Tay, 2013[121]	Singapore	No	Yes	NS	NS	NS	MP	100%	NS
Wu, 2013[122]	China	Yes	Yes	Inpatients with effusion	No definite diagnosis, chest trauma, prior invasive pleural intervention, receiving anti-TB or immunosuppressive drugs	None	CMP	80.0%	NS
Abrao, 2014[123]	Brazil	No	No	Inpatients with effusion with age >18 years	Prior treatment	NS	CMP	55.7%	NS
Anwar, 2014[124]	Pakistan	Yes	No	Age >12 years	Enteric fever, viral hepatitis or active cirrhosis in past	NS	CMP	NS	NS
Kong, 2014[125]	China	No	No	Patient undergoing diagnostic medical thoracoscopy	Incomplete histopathologic or clinical data	NS	MP	100%	Professional association
Li, 2014[126]	China	NS	Yes	Inpatients with effusion	NS	NS	MP	100%	NS
Liao, 2014[127]	China	Yes	No	NS	NS	None	MP	100%	Professional association
Mehta, 2014[128]	India	Yes	No	Exudative pleural effusion	Post-traumatic pleural effusion	NS	CMP	32.7%	NS
Meldau, 2014[129]	South Africa	Yes	No	Clinically suspected pleural tuberculosis	Incomplete clinical data, on anti-TB drugs for >48 hours	11/65	MP	100%	Professional association
Rahim, 2014[130]	Pakistan	Yes	Yes	Clinically suspected pleural tuberculosis, or transudative pleural effusion (controls)	NS	NS	CMP	NS	NS
Reis, 2014[131]	Portugal	No	No	Inpatients with effusion of unknown etiology	NS	NS	CMP	NS	NS
Sanchez-Otero, 2014[132]	Spain	NS	NS	NS	NS	NS	NS	NS	NS
Sethi, 2014[133]	India	Yes	No	NS	NS	NS	CMP	33.7%	Government
Trajman, 2014[134]	Brazil	Yes	No	Inpatients >18 years age with pleural effusion	Bleeding diathesis, inadequate fluid volume, no definite diagnosis	5/35	CMP	53.8%	NS
Valdes, 2014[135]	Spain	Yes	No	Inpatients with effusion	No definite diagnosis	NS	MP	100%	NS
Yurt, 2014[136]	Turkey	Yes	No	Inpatients with effusion	No definite diagnosis, transudates, patients on anti-TB drugs, HIV seropsitivity	NS	MP	100%	None

Agha, 2015[137]	Egypt	No	Yes	Inpatients with effusion	Hepatic or renal impairment, presence of pleural thickening, diagnosis from thoracoscopy	NS	P	100%	NS
Ali, 2015[138]	Egypt	Yes	Yes	Tuberculous or malignant pleural effusion	Patients on anti-TB or immunosuppressive drugs	NS	MP	100%	NS
Arnold, 2015[139]	UK	Yes	No	Undiagnosed pleural effusion	NS	None	CMP	85.7%	NS
Behrsin, 2015[140]	Brazil	Yes	No	NS	Hemolysis in pleural fluids, no definite diagnosis, renal failure or immunodeficiency disease	None	MP	100%	NS
Farhana, 2015[141]	Bangladesh	Yes	No	Inpatients with effusion	Hemothorax, empyema, patients on anti-TB drugs	NS	CMP	67.5%	NS
He, 2015[142]	China	Yes	No	Patients undergoing thoracoscopy	No definite diagnosis, absence of follow-up data	NS	MP	100%	Government
Klimiuk, 2015[143]	Poland	Yes	No	NS	No definite diagnosis, lack of consent for thoracocentesis	NS	CMP	NS	NS
Kosar, 2015[144]	Turkey	Yes	No	Inpatients with effusion	NS	NS	MP	100%	NS
Kumar, 2015[145]	India	Yes	No	Patients with advanced renal failure and pleural effusion undergoing thoracoscopy	Minimal pleural effusion, parapneumonic effusion, coagulopathy, patients not willing for thoracoscopy	None	MP	100%	NS
Li, 2015[146]	China	Yes	No	Inpatients with effusion	NS	None	CMP	71.9%	Professional association
Saiphoklang, 2015[147]	Thailand	No	Yes	Tuberculous or malignant effusion, age >15 years	NS	5/120	CMP	31.9%	Institutional
Shu, 2015[148]	Taiwan	Yes	No	Exudative and lymphocytic pleural effusion	HIV co-infection	NS	MP	100%	Professional association
Skouras, 2015[149]	Greece	No	No	Banked fluids from exudative pleural effusion	No definite diagnosis, transudates, hemothorax,	NS	CMP	80.0%	Professional association
Tural Onur, 2015[150]	Turkey	Yes	No	Inpatients with exudative pleural effusion	NS	NS	MP	100%	NS
Yoshino, 2015[151]	Japan	Yes	No	Infective pleurisy, age >18 years	Respiratory malignancy, rheumatoid arthritis	NS	CMP	NS	NS
Biswas, 2016[152]	India	Yes	No	Inpatients with effusion	Transudates, empyema	NS	NS	NS	None

Coral-Gudino, 2016[153]	Spain	No	Yes	Complicated tuberculous or parapneumonic effusion	Empyema	NS	CMP	40.1%	NS
Kim, 2016[154]	Korea	Yes	No	Inpatients with unilateral effusion suspected to be due to TB	No definite diagnosis	2/69	CMP	81.8%	Government
Lee, 2016[155]	Korea	No	Yes	Loculated neutrophilic pleural effusion due to TB or pneumonia	Empyema, no definite diagnosis	NS	MP	100%	NS
Liu, 2016[156]	China	Yes	Yes	Tuberculous or malignant effusion, age >12 years	Coexistent TB and malignancy	None	CMP	40.7%	Professional association
Mallik, 2016[157]	India	NS	No	NS	NS	NS	CMP	NS	None
Michot, 2016[158]	France	No	No	Adults with newly diagnosed pleural effusion and at least one ADA measurement	Empyema	5/104	CMP	61.8%	NS
Rahman, 2016[159]	India	No	No	Inpatients with effusion	NS	NS	CMP	NS	None
Saiphoklang, 2016[160]	Thailand	Yes	No	Age >=15 years	NS	NS	CMP	44.8%	Institutional
Suleman, 2016[161]	Pakistan	Yes	No	Lymphocytic exudative pleural effusion	NS	NS	P	100%	NS
Che, 2017[162]	China	Yes	No	Clinically suspected pleural tuberculosis	Incomplete clinical or outcome data	1/78	CMP	53.3%	Government
Chung, 2017[163]	Korea	Yes	No	Inpatients with effusion	Poor sample condition (such as clotting)	NS	CMP	84.0%	Institutional
El Hoshy, 2017[164]	Egypt	No	Yes	Inpatients with effusion	NS	NS	M	100%	NS
Kim, 2017[165]	Korea	No	Yes	Confirmed diagnosis of pleural effusion due to tuberculosis or Mycoplasma infection	Non-lymphocytic pleural effusion	NS	MP	100%	Government
Sivakumar, 2017[166]	UK	No	Yes	Adults with pleural effusion having ADA measurement	Suspected but unconfirmed pleural tuberculosis	None	MP	100%	NS
Xu, 2017[167]	China	Yes	No	Adults with unilateral pleural effusion	Immunosuppression, history of anti-tubercular therapy or previous tuberculosis	None	CMP	42.2%	Government
Zhang, 2017[168]	China	Yes	No	Adult inpatients with clinically suspected pleural tuberculosis	HIV co-infection, previous treatment for tuberculosis,	None	MP	100%	NS

					transudative effusion, no definite diagnosis				
Blakiston, 2018[169]	New Zealand	No	No	Adults with pleural effusion having ADA measurement	NS	NS	CMP	87.7%	NS
Chang, 2018[170]	Hong Kong	No	No	Exudative pleural effusion having ADA measurement	NS	NS	CMP	72.0%	NS
He, 2018[171]	China	Yes	No	Inpatients with undiagnosed pleural effusion	NS	NS	CMP	81.6%	Government
Hong, 2018[172]	Korea	Yes	No	Adults with pleural effusion	Hemothorax, parapneumonic effusion,	NS	CMP	63.8%	Government
Santos, 2018[173]	Brazil	Yes	No	Adults undergoing diagnostic thoracentesis	Pregnant women	4/147	CMP	48.5%	NS
Wang, 2018[174]	China	Yes	No	Inpatients with pleural effusion of definite etiology	NS	NS	MP	100%	Government

HIV+ Human immunodeficiency virus seropositivity, NS Not specified, TB Tuberculosis

Standard for diagnosis: C Clinical, M Microbiologic, P Pathologic

* Definite TB implies microbiologic and/or histopathologic confirmation of diagnosis in patients with tuberculous pleural effusion

See Table S1 for detailed bibliography
