# Supplementary material for: Adenosine deaminase for diagnosis of tuberculous pleural effusion: A systematic review and meta-analysis
Source: PLoS One. 2019 Mar 26;14(3):e0213728. doi: 10.1371/journal.pone.0213728 (PMC6435228; doi:10.1371/journal.pone.0213728)
Supplement: S3 Table — (PDF) [file pone.0213728.s007.pdf]

S3 Table. Clinical characteristics, and adenosine deaminase assay technique and results, from studies included in data synthesis.

Author, year	Patient number		Etiology of non-tubercular effusions			Assay method	Cut-off (IU/L)	TP	FN	TN	FP
	TB	Others	Malignant	Empyema / parapneumonic	Transudate						
Piras, 1978[1]	21	33	Yes	Yes	No	Guisti	30	21	0	33	0
Blake, 1982[2]	82	120	Yes	Yes	Yes	non-Guisti	30	74	8	118	2
Maritz, 1982[3]	107	237	Yes	Yes	Yes	Guisti	40	100	7	188	49
Petterson, 1984[4]	19	72	Yes	Yes	Yes	Guisti	50	19	0	62	10
Niwa, 1985[5]	30	28	Yes	No	No	NS	30	26	4	22	6
Raj, 1985[6]	30	25	Yes	Yes	Yes	Guisti	40	30	0	24	1
Sinha, 1987[7]	37	16	Yes	Yes	Yes	Guisti	30	37	0	16	0
Strankinga, 1987[8]	10	69	Yes	Yes	Yes	Guisti	53	10	0	60	9
Teo, 1987[9]	25	67	Yes	Yes	Yes	Guisti	50	24	1	61	6
van Keimpema, 1987[10]	5	93	Yes	Yes	Yes	non-Guisti	44	4	1	85	8
Fontan Bueso, 1988[11]	61	77	Yes	Yes	Yes	Guisti	33	61	0	68	9
Kao, 1988[12]	18	41	Yes	Yes	Yes	non-Guisti	60	18	0	36	5
Kim, 1988[13]	28	22	Yes	No	No	Guisti	41	25	3	19	3
Tamura, 1988[14]	8	44	Yes	Yes	Yes	non-Guisti	40	6	2	42	2
Gilhotra, 1989[15]	30	43	Yes	Yes	Yes	Guisti	40	30	0	39	4
Hsu, 1989[16]	19	20	Yes	Yes	No	Guisti	50	18	1	18	2
Moriwaki, 1989[17]	14	37	Yes	No	No	non-Guisti	33	14	0	35	2
Segura, 1989[18]	170	430	Yes	Yes	Yes	Guisti	42.6	170	0	395	35
Gourgoulianis, 1990[19]	37	83	Yes	Yes	No	Guisti	38	37	0	81	2
Gupta, 1990[20]	36	17	Yes	Yes	No	Guisti	50.75	36	0	16	1
Banales, 1991[21]	82	136	Yes	Yes	No	Guisti	70	81	1	130	6
Lopez Jimenez, 1991[22]	32	125	Yes	Yes	Yes	non-Guisti	32	28	4	114	11
Maartens, 1991[23]	61	48	Yes	No	Yes	NS	45	47	14	40	8

Hara, 1992[24]	26	36	Yes	Yes	No	NS	50	18	8	35	1
Kaur, 1992[25]	32	52	Yes	Yes	Yes	Guisti	30	16	16	48	4
Muranishi, 1992[26]	34	33	Yes	Yes	Yes	Guisti	50	16	18	25	8
Nagaraja, 1992[27]	15	15	Yes	Yes	Yes	Guisti	50	15	0	15	0
Perez-Rodriguez, 1992[28]	17	84	Yes	Yes	Yes	non-Guisti	40	15	2	83	1
Prasad, 1992[29]	21	26	Yes	Yes	Yes	non-Guisti	30	21	0	26	0
Hsu, 1993[30]	30	30	Yes	No	No	Guisti	60<br>80<br>100	24<br>24<br>22	6<br>6<br>8	26<br>27<br>27	4<br>3<br>3
Valdes, 1993[31]	91	314	Yes	Yes	Yes	Guisti	47	91	0	298	16
Aoki, 1994[32]	11	28	Yes	Yes	Yes	non-Guisti	45	9	2	25	3
Chiang, 1994[33]	27	66	Yes	Yes	Yes	Guisti	60	23	4	63	3
De Olivera, 1994[34]	54	222	Yes	Yes	Yes	Guisti	40	51	3	191	31
Richter, 1994[35]	112	6	Yes	Yes	No	NS	10	109	3	3	3
Burgess, 1995[36]	154	289	Yes	Yes	Yes	Guisti	50	139	15	257	32
Querol, 1995[37]	21	86	Yes	Yes	Yes	non-Guisti	45	18	3	84	2
Valdes, 1995[38]	81	48	Yes	Yes	No	Guisti	47	81	0	42	6
Orphanidou, 1996[39]	33	64	Yes	Yes	Yes	Guisti	40.6	26	7	60	4
Valdes, 1996[40]	76	274	Yes	Yes	Yes	Guisti	47	76	0	249	25
Villena, 1996[41]	49	179	Yes	Yes	Yes	non-Guisti	33	44	5	170	9
Ogawa, 1997[42]	16	25	Yes	Yes	No	NS	33	15	1	20	5
Kuralay, 1998[43]	30	59	Yes	Yes	Yes	non-Guisti	30	24	6	52	7
Ghelani, 1999[44]	54	27	Yes	Yes	Yes	Guisti	40	41	13	16	11
Perez-Rodriguez, 1999[45]	27	76	Yes	Yes	Yes	non-Guisti	40	24	3	70	6
Riantawan, 1999[46]	100	116	Yes	Yes	Yes	Guisti	60	95	5	97	19
San Jose, 1999[47]	23	117	Yes	Yes	Yes	Guisti	54	23	0	113	4
Gorguner, 2000[48]	36	51	Yes	Yes	Yes	Guisti	47	32	4	46	5

Prandaman, 2000[49]	25	12	Yes	No	Yes	Guisti	47.3	19	6	7	5
Villegas, 2000[50]	61	70	Yes	Yes	Yes	Guisti	45.5	53	8	60	10
Nagesh, 2001[51]	20	40	Yes	No	Yes	Guisti	50	11	9	22	18
Reechaipichitkul, 2001[52]	50	82	Yes	No	No	Guisti	48	40	10	66	16
Sharma, 2001[53]	48	27	Yes	No	Yes	Guisti	35<br>100	40<br>19	8<br>29	18<br>27	9<br>0
Yamada, 2001[54]	21	42	Yes	Yes	No	non-Guisti	45	18	3	37	5
Andreasyan, 2002[55]	47	22	Yes	Yes	No	non-Guisti	20	45	2	20	2
Jimenez, 2002[56]	16	68	Yes	Yes	No	NS	40	14	2	66	2
Diacon, 2003[57]	39	10	Yes	No	No	Guisti	50	37	2	9	1
Lima, 2003[58]	16	29	Yes	No	No	Guisti	40	11	5	21	8
Porcel, 2003[59]	104	272	Yes	No	No	non-Guisti	40	97	7	259	13
Tahhan, 2003[60]	24	38	Yes	Yes	No	Guisti	40	22	2	34	4
Chen, 2004[61]	63	147	Yes	Yes	No	non-Guisti	55.8	55	8	135	12
Ghanei, 2004[62]	17	71	Yes	Yes	Yes	Guisti	47	9	8	69	2
Poyraz, 2004[63]	15	30	Yes	No	Yes	Guisti	45	13	2	30	0
Gaga, 2005[64]	36	34	Yes	No	No	Guisti	50	35	1	33	1
Gao, 2005[65]	141	49	Yes	No	No	non-Guisti	40.3	116	25	43	6
Moon, 2005[66]	57	54	Yes	Yes	No	Guisti	45	46	11	51	3
Okamoto, 2005[67]	11	32	Yes	No	No	non-Guisti	32	11	0	31	1
Sharma, 2005[68]	35	17	Yes	No	No	Guisti	33	32	3	17	0
Tozkoparan, 2005[69]	26	29	Yes	Yes	Yes	NS	50	24	2	27	2
Celik, 2006[70]	17	28	Yes	Yes	No	Guisti	35.55	17	0	28	0
Mishra, 2006[71]	20	11	Yes	Yes	No	Guisti	38	16	4	7	4
Morimoto, 2006[72]	19	46	Yes	Yes	Yes	non-Guisti	50<br>57<br>58	15<br>15<br>14	4<br>4<br>5	44<br>45<br>45	2<br>1<br>1
Antonangelo, 2007[73]	182	144	Yes	No	No	Guisti	40	182	0	131	13

Ariga, 2007[74]	27	47	Yes	Yes	Yes	NS	40.7	22	5	43	4
Cok, 2007[75]	16	19	Yes	No	No	Guisti	40	12	4	19	0
Daniil, 2007[76]	12	60	Yes	Yes	No	Guisti	45	9	3	47	13
Lamsal, 2007[77]	29	13	Yes	Yes	No	Guisti	45	22	7	13	0
Moon, 2007[78]	26	27	Yes	Yes	No	Guisti	45	25	1	19	8
Neves, 2007[79]	104	111	Yes	Yes	Yes	Guisti	39	99	5	92	19
Trajman, 2007[80]	84	27	Yes	Yes	Yes	Guisti	36.6	76	8	26	1
Xue, 2007[81]	45	42	Yes	No	No	non-Guisti	40	36	9	37	5
Baba, 2008[82]	197	40	Yes	Yes	Yes	Guisti	30	186	11	38	2
Bandyopadhyay, 2008[83]	34	15	NS	NS	NS	Guisti	30	20	14	9	6
Krenke, 2008[84]	28	66	Yes	Yes	Yes	Guisti	40.3	28	0	62	4
Verma, 2008[85]	41	9	Yes	No	No	Guisti	36	41	0	7	2
Zaric, 2008[86]	54	67	Yes	No	No	Guisti	49	48	6	47	20
Chang, 2009[87]	108	280	Yes	Yes	No	NS	40<br>48<br>50	102<br>100<br>99	6<br>8<br>9	245<br>255<br>255	35<br>25<br>25
Dheda, 2009[88]	55	19	Yes	Yes	No	Guisti	13<br>30<br>47	55<br>52<br>49	0<br>3<br>6	7<br>13<br>13	12<br>6<br>6
Kupeli, 2009[89]	18	69	Yes	Yes	Yes	non-Guisti	40	14	4	63	6
Valdes, 2009[90]	39	57	Yes	Yes	No	Guisti	54.3	38	1	53	4
Zemlin, 2009[91]	365	514	Yes	Yes	Yes	Guisti	52.4	342	23	456	58
Ciledag, 2010[92]	14	56	Yes	Yes	Yes	NS	41.5	11	3	48	8
Gupta, 2010[93]	56	40	Yes	Yes	No	non-Guisti	40	56	0	39	1
Katiyar, 2010[94]	52	50	Yes	Yes	No	NS	38	47	5	43	7
Pandit, 2010[95]	22	50	Yes	No	No	Guisti	40<br>70	22<br>13	0<br>9	27<br>50	23<br>0
Porcel, 2010[96]	59	496	Yes	Yes	Yes	Guisti	35	55	4	449	47

Song, 2010[97]	5	115	Yes	Yes	No	Guisti	30<br>70	5<br>4	0<br>1	99<br>107	16<br>8
Valdes, 2010[98]	165	53	Yes	Yes	No	Guisti	35	165	0	42	11
Wu, 2010[99]	23	56	Yes	Yes	Yes	non-Guisti	19.5	22	1	45	11
Ambade, 2011[100]	48	33	Yes	No	No	Guisti	71	38	10	25	8
Bhutia, 2011[101]	35	19	Yes	No	Yes	Guisti	40	33	2	17	2
Kalantri, 2011[102]	154	50	Yes	Yes	Yes	Guisti	44.75	122	32	46	4
Liu, 2011[103]	24	42	Yes	No	No	Guisti	30	17	7	40	2
Ogata, 2011[104]	124	311	Yes	Yes	Yes	NS	36	106	18	265	46
Yildiz, 2011[105]	114	82	Yes	No	No	Guisti	31<br>55	114<br>99	0<br>15	36<br>71	46<br>11
Antonangelo, 2012[106]	130	29	Yes	No	No	Guisti	40	130	0	20	9
Cirak, 2012[107]	23	77	Yes	Yes	Yes	Guisti	40	19	4	74	3
Demirer, 2012[108]	157	91	Yes	Yes	Yes	NS	30<br>35<br>38<br>46	148<br>132<br>126<br>113	9<br>25<br>31<br>44	59<br>66<br>72<br>84	32<br>25<br>19<br>7
Devkota, 2012[109]	65	29	Yes	Yes	Yes	NS	40<br>42.19<br>70	59<br>59<br>41	6<br>6<br>24	22<br>24<br>24	7<br>5<br>5
Garcia-Zamalloa, 2012[110]	73	399	Yes	Yes	Yes	non-Guisti	40	65	8	370	29
Kashiwabara, 2012[111]	21	126	Yes	Yes	No	non-Guisti	50	18	3	100	26
Kumar, 2012[112]	67	16	Yes	Yes	No	non-Guisti	40	67	0	14	2
Pal, 2012[113]	70	180	NS	NS	NS	Guisti	60	70	0	180	0
Wang, 2012[114]	78	44	Yes	Yes	Yes	Guisti	40	73	5	40	4
Kelam, 2013[115]	39	18	Yes	Yes	No	Guisti	40	35	4	9	9
Keng, 2013[116]	31	57	NS	NS	NS	Guisti	15.5<br>40	26<br>14	5<br>17	50<br>56	7<br>1
Khan, 2013[117]	72	31	Yes	Yes	No	Guisti	16.65<br>58.6	62<br>13	10<br>59	23<br>31	8<br>0

Khow-Ean, 2013[118]	18	30	Yes	No	No	Guisti	17.5<br>24<br>40.5	16<br>14<br>9	2<br>4<br>9	22<br>24<br>30	8<br>6<br>0
Lee, 2013[119]	60	160	Yes	Yes	Yes	Guisti	28	53	7	147	13
Sahn, 2013[120]	548	423	Yes	Yes	Yes	Guisti	44	535	13	394	29
Tay, 2013[121]	80	80	Yes	Yes	No	Guisti	45	75	5	66	14
Wu, 2013[122]	40	41	Yes	Yes	Yes	Guisti	24.5	35	5	35	6
Abrao, 2014[123]	174	135	Yes	Yes	No	Guisti	29	157	17	115	20
Anwar, 2014[124]	164	56	Yes	Yes	Yes	Guisti	40	161	3	53	3
Kong, 2014[125]	76	15	Yes	No	No	NS	40	62	14	9	6
Li, 2014[126]	47	43	Yes	No	No	non-Guisti	39	36	11	36	7
Liao, 2014[127]	281	51	Yes	Yes	Yes	Guisti	35	156	125	44	7
Mehta, 2014[128]	49	73	Yes	Yes	No	NS	40	43	6	59	14
Meldau, 2014[129]	43	41	Yes	Yes	Yes	Guisti	30<br>48.85	32<br>23	11<br>20	38<br>39	3<br>2
Rahim, 2014[130]	60	20	No	No	Yes	NS	40	33	27	19	1
Reis, 2014[131]	20	87	Yes	Yes	No	Guisti	40.5	19	1	80	7
Sanchez-Otero, 2014[132]	30	108	Yes	Yes	No	Guisti	40	29	1	97	11
Sethi, 2014[133]	92	14	Yes	Yes	No	Guisti	52.4	67	25	10	4
Trajman, 2014[134]	39	23	Yes	No	Yes	NS	40	35	4	20	3
Valdes, 2014[135]	70	361	Yes	Yes	Yes	non-Guisti	56	69	1	337	24
Yurt, 2014[136]	43	50	Yes	Yes	No	Guisti	40.68	38	5	44	6
Agha, 2015[137]	30	25	Yes	No	Yes	NS	30.49	29	1	21	4
Ali, 2015[138]	20	20	Yes	No	No	Guisti	35.5	17	3	17	3
Arnold, 2015[139]	7	331	Yes	Yes	Yes	non-Guisti	35	6	1	301	30
Behrsin, 2015[140]	122	96	Yes	Yes	Yes	Guisti	40	98	24	83	13
Farhana, 2015[141]	40	24	Yes	Yes	No	NS	40	38	2	20	4
He, 2015[142]	56	80	Yes	Yes	No	non-Guisti	30	45	11	74	6

Klimiuk, 2015[143]	43	153	Yes	Yes	Yes	NS	40	38	5	142	11
Kosar, 2015[144]	50	51	Yes	Yes	No	Guisti	42	44	6	35	16
Kumar, 2015[145]	6	20	No	No	No	Guisti	40	4	2	18	2
Li, 2015[146]	32	55	Yes	Yes	Yes	non-Guisti	10.25	28	4	48	7
Saiphoklang, 2015[147]	47	73	Yes	No	No	NS	30	40	7	71	2
Shu, 2015[148]	35	60	Yes	Yes	No	Guisti	40	14	21	59	1
Skouras, 2015[149]	10	111	Yes	Yes	No	Guisti	31	9	1	89	22
Tural Onur, 2015[150]	52	68	Yes	Yes	No	Guisti	62.44	44	8	55	13
Yoshino, 2015[151]	10	17	No	Yes	No	NS	51.9	9	1	11	6
Biswas, 2016[152]	9	42	Yes	No	No	NS	40	7	2	40	2
Coral-Gudino, 2016[153]	135	266	No	Yes	No	non-Guisti	40	133	2	139	127
Kim, 2016[154]	26	28	Yes	Yes	Yes	NS	32<br>40	21<br>20	5<br>5	22<br>22	6<br>6
Lee, 2016[155]	10	54	No	Yes	No	non-Guisti	45.2	10	0	42	8
Liu, 2016[156]	91	26	Yes	No	No	NS	15.35	86	5	25	1
Mallik, 2016[157]	68	32	Yes	Yes	Yes	Guisti	40	66	2	31	1
Michot, 2016[158]	34	70	Yes	Yes	Yes	Guisti	41.5	33	1	65	5
Rahman, 2016[159]	84	16	Yes	NS	No	Guisti	50	84	0	14	2
Saiphoklang, 2016[160]	29	149	Yes	Yes	Yes	Guisti	33.5	27	2	141	8
Suleman, 2016[161]	99	61	Yes	Yes	No	Guisti	40	88	11	47	14
Che, 2017[162]	60	18	Yes	Yes	Yes	NS	40	41	19	16	2
Chung, 2017[163]	106	230	Yes	Yes	Yes	Guisti	46.1	93	13	217	13
El Hoshy, 2017[164]	20	40	Yes	Yes	No	non-Guisti	45	15	5	30	10
Kim, 2017[165]	275	8	No	Yes	No	NS	53	231	44	6	2
Sivakumar, 2017[166]	27	105	Yes	Yes	Yes	non-Guisti	30<br>35<br>40	26<br>24<br>23	1<br>3<br>4	88<br>90<br>93	17<br>15<br>12
Xu, 2017[167]	97	80	Yes	Yes	No	Guisti	45	60	37	76	4

Zhang, 2017[168]	354	84	Yes	Yes	No	non-Guisti	40	354	0	64	20
Blakiston, 2018[169]	57	1580	Yes	Yes	Yes	non-Guisti	15<br>30	57<br>53	0<br>4	991<br>1379	589<br>201
Chang, 2018[170]	152	474	Yes	Yes	No	non-Guisti	27.5	134	18	416	58
He, 2018[171]	49	60	Yes	Yes	Yes	non-Guisti	13.45	37	12	56	4
Hong, 2018[172]	47	28	Yes	Yes	Yes	non-Guisti	40<br>70	46<br>38	1<br>9	27<br>28	1<br>0
Santos, 2018[173]	33	46	Yes	Yes	Yes	Guisti	40	27	6	44	2
Wang, 2018[174]	95	179	Yes	Yes	No	non-Guisti	21.4	84	11	155	24

FN False negative, FP False positive, HIV+ Human immunodeficiency virus seropositivity, NS Not specified, TN True negative, TP True positive
See Table S1 for detailed bibliography
