# Supplementary material for: Adenosine deaminase for diagnosis of tuberculous pleural effusion: A systematic review and meta-analysis
Source: PLoS One. 2019 Mar 26;14(3):e0213728. doi: 10.1371/journal.pone.0213728 (PMC6435228; doi:10.1371/journal.pone.0213728)
Supplement: S4 Table — (PDF) [file pone.0213728.s008.pdf]

S4 Table. Diagnostic accuracy estimates from included studies

Author, year	Sensitivity	Specificity	Positive likelihood ratio	Negative likelihood ratio	Diagnostic odds ratio
Piras, 1978[1]	1.00 (0.84-1.00)	1.00 (0.89-1.00)	66.45 (4.24-1041.7)	0.02 (0.00-0.36)	2881.00 (55.08-150702)
Blake, 1982[2]	0.90 (0.82-0.96)	0.98 (0.94-1.00)	54.15 (13.67-214.4)	0.10 (0.05-0.19)	545.75 (112.8-2640.36)
Maritz, 1982[3]	0.93 (0.87-0.97)	0.79 (0.74-0.84)	4.52 (3.51-5.83)	0.08 (0.04-0.17)	54.81 (23.94-125.48)
Petterson, 1984[4]	1.00 (0.82-1.00)	0.86 (0.76-0.93)	6.78 (3.86-11.92)	0.03 (0.00-0.45)	232.14 (13.00-4144.68)
Niwa, 1985[5]	0.87 (0.69-0.96)	0.79 (0.59-0.92)	4.04 (1.96-8.33)	0.17 (0.07-0.43)	23.83 (5.96-95.37)
Raj, 1985[6]	1.00 (0.88-1.00)	0.96 (0.80-1.00)	17.05 (3.60-80.68)	0.02 (0.00-0.27)	996.33 (38.85-25554.3)
Sinha, 1987[7]	1.00 (0.91-1.00)	1.00 (0.79-1.00)	33.55 (2.19-514.97)	0.01 (0.00-0.21)	2475.00 (47.07-130152)
Strankinga, 1987[8]	1.00 (0.69-1.00)	0.87 (0.77-0.94)	7.03 (3.84-12.88)	0.05 (0.00-0.79)	133.74 (7.23-2475.51)
Teo, 1987[9]	0.96 (0.80-1.00)	0.91 (0.82-0.97)	10.72 (4.98-23.1)	0.04 (0.01-0.30)	244.00 (27.89-2134.97)
van Keimpema, 1987[10]	0.80 (0.28-0.99)	0.91 (0.84-0.96)	9.30 (4.20-20.58)	0.22 (0.04-1.26)	42.50 (4.23-427.34)
Fontan Bueso, 1988[11]	1.00 (0.94-1.00)	0.88 (0.79-0.95)	8.14 (4.49-14.79)	0.01 (0.00-0.15)	886.89 (50.56-15557.6)
Kao, 1988[12]	1.00 (0.81-1.00)	0.88 (0.74-0.96)	7.44 (3.40-16.26)	0.03 (0.00-0.47)	245.55 (12.87-4684.83)
Kim, 1988[13]	0.89 (0.72-0.98)	0.86 (0.65-0.97)	6.55 (2.27-18.89)	0.12 (0.04-0.37)	52.78 (9.57-291.18)
Tamura, 1988[14]	0.75 (0.35-0.97)	0.95 (0.85-0.99)	16.50 (4.02-67.71)	0.26 (0.08-0.87)	63.00 (7.42-534.66)
Gilhotra, 1989[15]	1.00 (0.88-1.00)	0.91 (0.78-0.97)	9.62 (4.23-11.00)	0.02 (0.00-0.28)	535.44 (27.75-10329.8)
Hsu, 1989[16]	0.95 (0.74-1.00)	0.90 (0.68-0.99)	9.47 (2.53-35.43)	0.06 (0.01-0.40)	162.00 (13.46-1949.58)
Moriwaki, 1989[17]	1.00 (0.77-1.00)	0.95 (0.82-0.99)	14.69 (4.42-48.87)	0.04 (0.00-0.55)	411.80 (18.6-9115.54)
Segura, 1989[18]	1.00 (0.98-1.00)	0.92 (0.89-0.94)	12.11 (8.83-16.59)	0.00 (0.00-0.05)	3799.03 (231.71-62288)
Gourgoulianis, 1990[19]	1.00 (0.91-1.00)	0.98 (0.92-1.00)	33.16 (9.77-112.49)	0.01 (0.00-0.21)	2445.00 (114.54-52193)
Gupta, 1990[20]	1.00 (0.90-1.00)	0.94 (0.71-1.00)	11.84 (2.56-54.81)	0.01 (0.00-0.23)	803.00 (31.04-20771.7)
Banales, 1991[21]	0.99 (0.93-1.00)	0.96 (0.91-0.98)	22.39 (10.24-48.97)	0.01 (0.00-0.09)	1755.00 (207.5-14843.7)
Lopez Jimenez, 1991[22]	0.88 (0.71-0.96)	0.91 (0.85-0.96)	9.94 (5.57-17.75)	0.14 (0.05-0.34)	72.55 (21.49-244.93)
Maartens, 1991[23]	0.77 (0.65-0.87)	0.83 (0.70-0.93)	4.62 (2.42-8.83)	0.28 (0.17-0.44)	16.79 (6.39-44.08)
Hara, 1992[24]	0.69 (0.48-0.86)	0.97 (0.85-1.00)	24.92 (3.55-175.08)	0.32 (0.18-0.56)	78.75 (9.13-679.57)

Kaur, 1992[25]	0.50 (0.32-0.68)	0.92 (0.81-0.98)	6.50 (2.38-17.73)	0.54 (0.38-0.77)	12.00 (3.50-41.18)
Muranishi, 1992[26]	0.47 (0.30-0.65)	0.76 (0.58-0.89)	1.94 (0.96-3.91)	0.70 (0.48-1.01)	2.78 (0.98-7.88)
Nagaraja, 1992[27]	1.00 (0.78-1.00)	1.00 (0.78-1.00)	31.00 (2.02-475.12)	0.03 (0.00-0.49)	961.00 (17.91-51566.4)
Perez-Rodriguez, 1992[28]	0.88 (0.64-0.99)	0.99 (0.94-1.00)	74.12 (10.48-524.1)	0.12 (0.03-0.44)	622.50 (53.04-7305.47)
Prasad, 1992[29]	1.00 (0.84-1.00)	1.00 (0.87-1.00)	52.77 (3.38-822.84)	0.02 (0.00-0.36)	2279.00 (43.40-119669)
Hsu, 1993[30]	0.80 (0.61-0.92)	0.90 (0.73-0.98)	8.00 (2.69-23.75)	0.22 (0.11-0.46)	36.00 (8.11-159.89)
Valdes, 1993[31]	1.00 (0.96-1.00)	0.95 (0.92-0.97)	18.99 (11.87-30.38)	0.01 (0.00-0.09)	3310.64 (196.7-55721.3)
Aoki, 1994[32]	0.82 (0.48-0.98)	0.89 (0.72-0.98)	7.64 (2.53-23.05)	0.20 (0.06-0.72)	37.50 (5.36-262.17)
Chiang, 1994[33]	0.85 (0.66-0.96)	0.95 (0.87-0.99)	18.74 (6.13-57.25)	0.16 (0.06-0.38)	120.75 (25.09-581.13)
De Olivera, 1994[34]	0.94 (0.85-0.99)	0.86 (0.81-0.9)	6.76 (4.85-9.43)	0.06 (0.02-0.19)	104.74 (30.78-356.44)
Richter, 1994[35]	0.97 (0.92-0.99)	0.50 (0.12-0.88)	1.95 (0.87-4.34)	0.05 (0.01-0.21)	36.33 (5.07-260.26)
Burgess, 1995[36]	0.90 (0.84-0.94)	0.89 (0.85-0.92)	8.15 (5.86-11.35)	0.11 (0.07-0.18)	74.42 (38.97-142.15)
Querol, 1995[37]	0.86 (0.64-0.97)	0.98 (0.92-1.00)	36.86 (9.27-146.62)	0.15 (0.05-0.42)	252.00 (39.22-1619.11)
Valdes, 1995[38]	1.00 (0.96-1.00)	0.88 (0.75-0.95)	7.49 (3.66-15.33)	0.01 (0.00-0.11)	1065.77 (58.63-19374.3)
Orphanidou, 1996[39]	0.79 (0.61-0.91)	0.94 (0.85-0.98)	12.61 (4.80-33.10)	0.23 (0.12-0.44)	55.71 (15.01-206.87)
Valdes, 1996[40]	1.00 (0.95-1.00)	0.91 (0.87-0.94)	10.71 (7.40-15.51)	0.01 (0.00-0.11)	1497.00 (90.08-24878.6)
Villena, 1996[41]	0.90 (0.78-0.97)	0.95 (0.91-0.98)	17.86 (9.38-33.99)	0.11 (0.05-0.25)	166.22 (53.03-520.97)
Ogawa, 1997[42]	0.94 (0.70-1.00)	0.80 (0.59-0.93)	4.69 (2.12-10.37)	0.08 (0.01-0.53)	60.00 (6.33-568.69)
Kuralay, 1998[43]	0.80 (0.61-0.92)	0.88 (0.77-0.95)	6.74 (3.29-13.83)	0.23 (0.11-0.47)	29.71 (9.01-97.95)
Ghelani, 1999[44]	0.76 (0.62-0.87)	0.59 (0.39-0.78)	1.86 (1.15-3.01)	0.41 (0.23-0.72)	4.59 (1.71-12.34)
Perez-Rodriguez, 1999[45]	0.89 (0.71-0.98)	0.92 (0.84-0.97)	11.26 (5.16-24.55)	0.12 (0.04-0.35)	93.33 (21.65-402.45)
Riantawan, 1999[46]	0.95 (0.89-0.98)	0.84 (0.76-0.90)	5.80 (3.84-8.77)	0.06 (0.03-0.14)	97.00 (34.81-270.33)
San Jose, 1999[47]	1.00 (0.85-1.00)	0.97 (0.91-0.99)	25.68 (10.36-63.66)	0.02 (0.00-0.34)	1185.44 (61.72-22769.7)
Gorguner, 2000[48]	0.89 (0.74-0.97)	0.90 (0.79-0.97)	9.07 (3.91-21.01)	0.12 (0.05-0.31)	73.60 (18.33-295.5)
Prandaman, 2000[49]	0.76 (0.55-0.91)	0.58 (0.28-0.85)	1.82 (0.90-3.69)	0.41 (0.18-0.96)	4.43 (1.02-19.27)
Villegas, 2000[50]	0.87 (0.76-0.94)	0.86 (0.75-0.93)	6.08 (3.40-10.88)	0.15 (0.08-0.29)	39.75 (14.62-108.1)

Nagesh, 2001[51]	0.55 (0.32-0.77)	0.55 (0.38-0.71)	1.22 (0.72-2.06)	0.82 (0.47-1.43)	1.49 (0.51-4.39)
Reechaipichitkul, 2001[52]	0.80 (0.66-0.90)	0.80 (0.70-0.88)	4.10 (2.59-6.5)	0.25 (0.14-0.44)	16.50 (6.83-39.87)
Sharma, 2001[53]	0.83 (0.70-0.93)	0.67 (0.46-0.83)	2.50 (1.44-4.33)	0.25 (0.13-0.50)	10.00 (3.32-30.13)
Yamada, 2001[54]	0.86 (0.64-0.97)	0.88 (0.74-0.96)	7.20 (3.11-16.69)	0.16 (0.06-0.47)	44.40 (9.54-206.73)
Andreasyan, 2002[55]	0.96 (0.85-0.99)	0.91 (0.71-0.99)	10.53 (2.81-39.54)	0.05 (0.01-0.18)	225.00 (29.57-1712.32)
Jimenez, 2002[56]	0.88 (0.62-0.98)	0.97 (0.90-1.00)	29.75 (7.50-118)	0.13 (0.04-0.47)	231.00 (29.95-1781.96)
Diacon, 2003[57]	0.95 (0.83-0.99)	0.90 (0.55-1.00)	9.49 (1.48-60.99)	0.06 (0.01-0.22)	166.50 (13.55-2045.81)
Lima, 2003[58]	0.69 (0.41-0.89)	0.72 (0.53-0.87)	2.49 (1.27-4.9)	0.43 (0.20-0.92)	5.78 (1.52-21.93)
Porcel, 2003[59]	0.93 (0.87-0.97)	0.95 (0.92-0.97)	19.51 (11.45-33.25)	0.07 (0.03-0.14)	276.08 (106.98-712.43)
Tahhan, 2003[60]	0.92 (0.73-0.99)	0.89 (0.75-0.97)	8.71 (3.42-22.18)	0.09 (0.02-0.35)	93.50 (15.77-554.49)
Chen, 2004[61]	0.87 (0.77-0.94)	0.92 (0.86-0.96)	10.69 (6.17-18.54)	0.14 (0.07-0.26)	77.34 (29.97-199.58)
Ghanei, 2004[62]	0.53 (0.28-0.77)	0.97 (0.90-1.00)	18.79 (4.46-79.16)	0.48 (0.29-0.80)	38.81 (7.10-212.05)
Poyraz, 2004[63]	0.87 (0.60-0.98)	1.00 (0.88-1.00)	52.31 (3.32-824.41)	0.16 (0.05-0.50)	329.40 (14.79-7335.62)
Gaga, 2005[64]	0.97 (0.85-1.00)	0.97 (0.85-1.00)	33.06 (4.79-228.13)	0.03 (0.00-0.20)	1155.00 (69.38-19228.8)
Gao, 2005[65]	0.82 (0.75-0.88)	0.88 (0.75-0.95)	6.72 (3.16-14.27)	0.20 (0.14-0.29)	33.25 (12.77-86.61)
Moon, 2005[66]	0.81 (0.68-0.90)	0.94 (0.85-0.99)	14.53 (4.80-43.95)	0.20 (0.12-0.35)	71.09 (18.66-270.78)
Okamoto, 2005[67]	1.00 (0.72-1.00)	0.97 (0.84-1.00)	21.08 (4.40-101.13)	0.04 (0.00-0.66)	483.00 (18.34-12721.9)
Sharma, 2005[68]	0.91 (0.77-0.98)	1.00 (0.80-1.00)	32.50 (2.11-500.89)	0.10 (0.04-0.27)	325.00 (15.87-6657.23)
Tozkoparan, 2005[69]	0.92 (0.75-0.99)	0.93 (0.77-0.99)	13.38 (3.50-51.21)	0.08 (0.02-0.31)	162.00 (21.16-1240.45)
Celik, 2006[70]	1.00 (0.80-1.00)	1.00 (0.88-1.00)	56.39 (3.61-881.12)	0.03 (0.00-0.43)	1995.00 (37.85-105162)
Mishra, 2006[71]	0.80 (0.56-0.94)	0.64 (0.31-0.89)	2.20 (0.98-4.95)	0.31 (0.12-0.84)	7.00 (1.35-36.31)
Morimoto, 2006[72]	0.79 (0.54-0.94)	0.98 (0.88-1.00)	36.32 (5.15-255.87)	0.22 (0.09-0.51)	168.75 (17.47-1629.94)
Antonangelo, 2007[73]	1.00 (0.98-1.00)	0.91 (0.85-0.95)	10.71 (6.44-17.8)	0.00 (0.00-0.05)	3555.37 (209.49-60341)
Ariga, 2007[74]	0.81 (0.62-0.94)	0.91 (0.80-0.98)	9.57 (3.69-24.87)	0.20 (0.09-0.45)	47.30 (11.53-194.05)
Cok, 2007[75]	0.75 (0.48-0.93)	1.00 (0.82-1.00)	29.41 (1.88-460.89)	0.27 (0.12-0.60)	108.33 (5.36-2190.80)
Daniil, 2007[76]	0.75 (0.43-0.95)	0.78 (0.66-0.88)	3.46 (1.94-6.19)	0.32 (0.12-0.86)	10.85 (2.56-45.95)

Lamsal, 2007[77]	0.76 (0.56-0.90)	1.00 (0.75-1.00)	21.00 (1.37-321.89)	0.26 (0.14-0.49)	81.00 (4.28-1533.96)
Moon, 2007[78]	0.96 (0.80-1.00)	0.70 (0.50-0.86)	3.25 (1.81-5.83)	0.05 (0.01-0.38)	59.38 (6.83-516.27)
Neves, 2007[79]	0.95 (0.89-0.98)	0.83 (0.75-0.89)	5.56 (3.68-8.39)	0.06 (0.02-0.14)	95.87 (34.39-267.26)
Trajman, 2007[80]	0.90 (0.82-0.96)	0.96 (0.81-1.00)	24.43 (3.57-167.39)	0.10 (0.05-0.19)	247.00 (29.47-2070.20)
Xue, 2007[81]	0.80 (0.65-0.90)	0.88 (0.74-0.96)	6.72 (2.91-15.5)	0.23 (0.13-0.41)	29.60 (9.04-96.87)
Baba, 2008[82]	0.94 (0.90-0.97)	0.95 (0.83-0.99)	18.88 (4.89-72.93)	0.06 (0.03-0.10)	321.27 (68.43-1508.4)
Bandyopadhyay, 2008[83]	0.59 (0.41-0.75)	0.60 (0.32-0.84)	1.47 (0.74-2.9)	0.69 (0.39-1.22)	2.14 (0.62-7.39)
Krenke, 2008[84]	1.00 (0.88-1.00)	0.94 (0.85-0.98)	14.63 (5.99-35.76)	0.02 (0.00-0.29)	791.67 (41.22-15203.4)
Verma, 2008[85]	1.00 (0.91-1.00)	0.78 (0.40-0.97)	3.95 (1.35-11.57)	0.02 (0.00-0.26)	249.00 (10.84-5720.55)
Zaric, 2008[86]	0.89 (0.77-0.96)	0.70 (0.58-0.81)	2.98 (2.04-4.35)	0.16 (0.07-0.34)	18.80 (6.94-50.95)
Chang, 2009[87]	0.93 (0.86-0.97)	0.91 (0.87-0.94)	10.37 (7.11-15.13)	0.08 (0.04-0.16)	127.50 (55.65-292.12)
Dheda, 2009[88]	0.95 (0.85-0.99)	0.68 (0.43-0.87)	2.99 (1.54-5.82)	0.08 (0.03-0.25)	37.56 (8.27-170.57)
Kupeli, 2009[89]	0.78 (0.52-0.94)	0.91 (0.82-0.97)	8.94 (4.01-19.98)	0.24 (0.1-0.58)	36.75 (9.14-147.75)
Valdes, 2009[90]	0.97 (0.87-1.00)	0.93 (0.83-0.98)	13.88 (5.39-35.77)	0.03 (0.00-0.19)	503.50 (54.11-4685.10)
Zemlin, 2009[91]	0.94 (0.91-0.96)	0.89 (0.86-0.91)	8.30 (6.51-10.6)	0.07 (0.05-0.11)	116.91 (70.7-193.30)
Ciledag, 2010[92]	0.79 (0.49-0.95)	0.86 (0.74-0.94)	5.50 (2.74-11.05)	0.25 (0.09-0.69)	22.00 (5.01-96.63)
Gupta, 2010[93]	1.00 (0.94-1.00)	0.98 (0.87-1.00)	27.09 (5.63-130.35)	0.01 (0.00-0.14)	2975.67 (118.14-74947)
Katiyar, 2010[94]	0.90 (0.79-0.97)	0.86 (0.73-0.94)	6.46 (3.23-12.91)	0.11 (0.05-0.26)	57.74 (17.05-195.57)
Pandit, 2010[95]	0.59 (0.36-0.79)	1.00 (0.93-1.00)	59.87 (3.72-964.47)	0.42 (0.26-0.68)	143.53 (7.85-2625.55)
Porcel, 2010[96]	0.93 (0.84-0.98)	0.91 (0.88-0.93)	9.84 (7.43-13.02)	0.07 (0.03-0.19)	131.36 (45.58-378.59)
Song, 2010[97]	1.00 (0.48-1.00)	0.86 (0.78-0.92)	6.44 (3.88-10.71)	0.10 (0.01-1.38)	66.33 (3.5-1256.71)
Valdes, 2010[98]	1.00 (0.98-1.00)	0.79 (0.66-0.89)	4.68 (2.80-7.82)	0.00 (0.00-0.06)	1223.26 (70.66-21177.4)
Wu, 2010[99]	0.96 (0.78-1.00)	0.80 (0.68-0.90)	4.87 (2.85-8.33)	0.05 (0.01-0.37)	90.00 (10.92-742.09)
Ambade, 2011[100]	0.79 (0.65-0.90)	0.76 (0.58-0.89)	3.27 (1.76-6.07)	0.28 (0.15-0.49)	11.88 (4.12-34.2)
Bhutia, 2011[101]	0.94 (0.81-0.99)	0.89 (0.67-0.99)	8.96 (2.41-33.31)	0.06 (0.02-0.25)	140.25 (18.14-1084.49)
Kalantri, 2011[102]	0.79 (0.72-0.85)	0.92 (0.81-0.98)	9.90 (3.85-25.44)	0.23 (0.16-0.31)	43.84 (14.69-130.84)

Liu, 2011[103]	0.71 (0.49-0.87)	0.95 (0.84-0.99)	14.87 (3.75-58.93)	0.31 (0.16-0.57)	48.57 (9.14-258.22)
Ogata, 2011[104]	0.85 (0.78-0.91)	0.85 (0.81-0.89)	5.78 (4.38-7.62)	0.17 (0.11-0.26)	33.93 (18.81-61.18)
Yildiz, 2011[105]	0.87 (0.79-0.92)	0.87 (0.77-0.93)	6.47 (3.72-11.27)	0.15 (0.09-0.25)	42.60 (18.47-98.24)
Antonangelo, 2012[106]	1.00 (0.97-1.00)	0.69 (0.49-0.85)	3.15 (1.86-5.32)	0.01 (0.00-0.09)	563.21 (31.56-10050.1)
Cirak, 2012[107]	0.83 (0.61-0.95)	0.96 (0.89-0.99)	21.20 (6.88-65.31)	0.18 (0.07-0.44)	117.17 (24.14-568.58)
Demirer, 2012[108]	0.72 (0.64-0.79)	0.92 (0.85-0.97)	9.36 (4.56-19.19)	0.30 (0.23-0.39)	30.82 (13.22-71.82)
Devkota, 2012[109]	0.91 (0.81-0.97)	0.83 (0.64-0.94)	5.26 (2.36-11.73)	0.11 (0.05-0.24)	47.20 (13.15-169.45)
Garcia-Zamalloa, 2012[110]	0.89 (0.80-0.95)	0.93 (0.90-0.95)	12.25 (8.55-17.55)	0.12 (0.06-0.23)	103.66 (45.39-236.76)
Kashiwabara, 2012[111]	0.86 (0.64-0.97)	0.79 (0.71-0.86)	4.15 (2.83-6.1.00)	0.18 (0.06-0.52)	23.08 (6.31-84.35)
Kumar, 2012[112]	1.00 (0.95-1.00)	0.88 (0.62-0.98)	6.75 (2.15-21.21)	0.01 (0.00-0.14)	783.00 (35.67-17189.2)
Pal, 2012[113]	1.00 (0.95-1.00)	1.00 (0.98-1.00)	359.45 (22.57-5725)	0.01 (0.00-0.11)	50901.0 (1000-2590130)
Wang, 2012[114]	0.94 (0.86-0.98)	0.91 (0.78-0.97)	10.29 (4.04-26.25)	0.07 (0.03-0.17)	146.00 (37.09-574.65)
Kelam, 2013[115]	0.90 (0.76-0.97)	0.50 (0.26-0.74)	1.79 (1.12-2.88)	0.21 (0.07-0.58)	8.75 (2.19-35.02)
Keng, 2013[116]	0.84 (0.66-0.95)	0.88 (0.76-0.95)	6.83 (3.36-13.9)	0.18 (0.08-0.41)	37.14 (10.73-128.56)
Khan, 2013[117]	0.86 (0.76-0.93)	0.74 (0.55-0.88)	3.34 (1.82-6.1.00)	0.19 (0.10-0.35)	17.83 (6.27-50.71)
Khow-Ean, 2013[118]	0.89 (0.65-0.99)	0.73 (0.54-0.88)	3.33 (1.80-6.17)	0.15 (0.04-0.57)	22.00 (4.11-117.80)
Lee, 2013[119]	0.88 (0.77-0.95)	0.92 (0.87-0.96)	10.87 (6.40-18.45)	0.13 (0.06-0.26)	85.62 (32.42-226.08)
Sahn, 2013[120]	0.98 (0.96-0.99)	0.93 (0.90-0.95)	14.24 (10.02-20.24)	0.03 (0.01-0.04)	559.12 (286.97-1089.4)
Tay, 2013[121]	0.94 (0.86-0.98)	0.82 (0.72-0.90)	5.36 (3.32-8.65)	0.08 (0.03-0.18)	70.71 (24.17-206.85)
Wu, 2013[122]	0.88 (0.73-0.96)	0.85 (0.71-0.94)	5.98 (2.83-12.64)	0.15 (0.06-0.34)	40.83 (11.40-146.27)
Abrao, 2014[123]	0.90 (0.85-0.94)	0.85 (0.78-0.91)	6.09 (4.05-9.15)	0.11 (0.07-0.18)	53.10 (26.64-105.86)
Anwar, 2014[124]	0.98 (0.95-1.00)	0.95 (0.85-0.99)	18.33 (6.09-55.11)	0.02 (0.01-0.06)	948.11 (185.74-4839.6)
Kong, 2014[125]	0.82 (0.71-0.90)	0.60 (0.32-0.84)	2.04 (1.09-3.83)	0.31 (0.16-0.58)	6.64 (2.03-21.72)
Li, 2014[126]	0.77 (0.62-0.88)	0.84 (0.69-0.93)	4.71 (2.35-9.44)	0.28 (0.16-0.48)	16.83 (5.86-48.3)
Liao, 2014[127]	0.56 (0.49-0.61)	0.86 (0.74-0.94)	4.04 (2.02-8.11)	0.52 (0.43-0.61)	7.84 (3.42-18.02)
Mehta, 2014[128]	0.88 (0.75-0.95)	0.81 (0.70-0.89)	4.58 (2.82-7.41)	0.15 (0.07-0.32)	30.20 (10.74-84.93)

Meldau, 2014[129]	0.74 (0.59-0.86)	0.93 (0.80-0.98)	10.17 (3.37-30.66)	0.28 (0.16-0.46)	36.85 (9.45-143.63)
Rahim, 2014[130]	0.55 (0.42-0.68)	0.95 (0.75-1.00)	11.00 (1.61-75.33)	0.47 (0.35-0.64)	23.22 (2.92-184.81)
Reis, 2014[131]	0.95 (0.75-1.00)	0.92 (0.84-0.97)	11.81 (5.76-24.2)	0.05 (0.01-0.37)	217.14 (25.19-1871.93)
Sanchez-Otero, 2014[132]	0.97 (0.83-1.00)	0.90 (0.83-0.95)	9.49 (5.40-16.68)	0.04 (0.01-0.26)	255.73 (31.67-2064.89)
Sethi, 2014[133]	0.73 (0.63-0.82)	0.71 (0.42-0.92)	2.55 (1.10-5.89)	0.38 (0.24-0.61)	6.70 (1.92-23.32)
Trajman, 2014[134]	0.90 (0.76-0.97)	0.87 (0.66-0.97)	6.88 (2.38-19.87)	0.12 (0.05-0.30)	58.33 (11.84-287.36)
Valdes, 2014[135]	0.99 (0.92-1.00)	0.93 (0.90-0.96)	14.83 (10.06-21.85)	0.02 (0.00-0.11)	968.88 (128.90-7282.4)
Yurt, 2014[136]	0.88 (0.75-0.96)	0.88 (0.76-0.95)	7.36 (3.45-15.72)	0.13 (0.06-0.30)	55.73 (15.75-197.21)
Agha, 2015[137]	0.97 (0.83-1.00)	0.84 (0.64-0.95)	6.04 (2.45-14.87)	0.04 (0.01-0.27)	152.25 (15.85-1462.13)
Ali, 2015[138]	0.85 (0.62-0.97)	0.85 (0.62-0.97)	5.67 (1.96-16.35)	0.18 (0.06-0.51)	32.11 (5.66-182.18)
Arnold, 2015[139]	0.86 (0.42-1.00)	0.91 (0.87-0.94)	9.46 (5.99-14.92)	0.16 (0.03-0.96)	60.20 (7.01-516.81)
Behrsin, 2015[140]	0.80 (0.72-0.87)	0.86 (0.78-0.93)	5.93 (3.55-9.91)	0.23 (0.16-0.33)	26.07 (12.49-54.40)
Farhana, 2015[141]	0.95 (0.83-0.99)	0.83 (0.63-0.95)	5.70 (2.32-13.98)	0.06 (0.02-0.23)	95.00 (15.99-564.26)
He, 2015[142]	0.80 (0.68-0.90)	0.93 (0.84-0.97)	10.71 (4.91-23.38)	0.21 (0.12-0.36)	50.45 (17.45-145.85)
Klimiuk, 2015[143]	0.88 (0.75-0.96)	0.93 (0.88-0.96)	12.29 (6.89-21.94)	0.13 (0.05-0.29)	98.11 (32.14-299.52)
Koser, 2015[144]	0.88 (0.76-0.95)	0.69 (0.54-0.81)	2.80 (1.85-4.26)	0.17 (0.08-0.38)	16.04 (5.68-45.29)
Kumar, 2015[145]	0.67 (0.22-0.96)	0.90 (0.68-0.99)	6.67 (1.59-27.9)	0.37 (0.12-1.16)	18.00 (1.92-168.99)
Li, 2015[146]	0.88 (0.71-0.96)	0.87 (0.76-0.95)	6.88 (3.40-13.9)	0.14 (0.06-0.36)	48.00 (12.9-178.59)
Saiphoklang, 2015[147]	0.85 (0.72-0.94)	0.97 (0.90-1.00)	31.06 (7.88-122.49)	0.15 (0.08-0.30)	202.86 (40.2-1023.55)
Shu, 2015[148]	0.40 (0.24-0.58)	0.98 (0.91-1.00)	24.00 (3.30-174.78)	0.61 (0.46-0.80)	39.33 (4.87-317.69)
Skouras, 2015[149]	0.90 (0.55-1.00)	0.80 (0.72-0.87)	4.54 (2.96-6.96)	0.12 (0.02-0.80)	36.41 (4.38-302.73)
Tural Onur, 2015[150]	0.85 (0.72-0.93)	0.81 (0.70-0.89)	4.43 (2.68-7.32)	0.19 (0.10-0.36)	23.27 (8.86-61.13)
Yoshino, 2015[151]	0.90 (0.55-1.00)	0.65 (0.38-0.86)	2.55 (1.30-5.01)	0.15 (0.02-1.03)	16.50 (1.67-163.42)
Biswas, 2016[152]	0.78 (0.40-0.97)	0.95 (0.84-0.99)	16.33 (4.04-66.03)	0.23 (0.07-0.79)	70.00 (8.42-582.06)
Coral-Gudino, 2016[153]	0.99 (0.95-1.00)	0.52 (0.46-0.58)	2.06 (1.82-2.34)	0.03 (0.01-0.11)	72.78 (17.65-300.17)
Kim, 2016[154]	0.81 (0.61-0.93)	0.79 (0.59-0.92)	3.77 (1.81-7.85)	0.24 (0.11-0.55)	15.40 (4.08-58.17)

Lee, 2016[155]	1.00 (0.69-1.00)	0.84 (0.71-0.93)	5.73 (3.06-10.72)	0.05 (0.00-0.82)	105.00 (5.60-1968.48)
Liu, 2016[156]	0.95 (0.88-0.98)	0.96 (0.80-1.00)	24.57 (3.59-168.03)	0.06 (0.02-0.13)	430.00 (47.99-3852.66)
Mallik, 2016[157]	0.97 (0.90-1.00)	0.97 (0.84-1.00)	31.06 (4.51-213.88)	0.03 (0.01-0.12)	1023.00 (89.34-11714.6)
Michot, 2016[158]	0.97 (0.85-1.00)	0.93 (0.84-0.98)	13.59 (5.83-31.69)	0.03 (0.00-0.22)	429.00 (48.13-3823.72)
Rahman, 2016[159]	1.00 (0.96-1.00)	0.88 (0.62-0.98)	6.76 (2.15-21.24)	0.01 (0.00-0.11)	980.20 (44.73-21478.5)
Saiphoklang, 2016[160]	0.93 (0.77-0.93)	0.95 (0.90-0.98)	17.34 (8.77-34.27)	0.07 (0.02-0.28)	237.94 (47.88-1182.36)
Suleman, 2016[161]	0.89 (0.81-0.94)	0.77 (0.65-0.87)	3.87 (2.43-6.17)	0.14 (0.08-0.26)	26.86 (11.30-63.81)
Che, 2017[162]	0.68 (0.55-0.80)	0.89 (0.65-0.99)	6.15 (1.65-22.97)	0.36 (0.24-0.53)	17.26 (3.60-82.76)
Chung, 2017[163]	0.88 (0.80-0.93)	0.94 (0.91-0.97)	15.52 (9.11-26.45)	0.13 (0.08-0.22)	119.41 (53.32-267.42)
El Hoshy, 2017[164]	0.75 (0.51-0.91)	0.75 (0.59-0.87)	3.00 (1.66-5.43)	0.33 (0.15-0.73)	9.00 (2.61-31.09)
Kim, 2017[165]	0.84 (0.79-0.88)	0.75 (0.35-0.97)	3.36 (1.01-11.17)	0.21 (0.13-0.35)	15.75 (3.08-80.58)
Sivakumar, 2017[166]	0.96 (0.81-1.00)	0.84 (0.75-0.90)	5.95 (3.83-9.25)	0.04 (0.01-0.30)	134.59 (17.09-1000.00)
Xu, 2017[167]	0.62 (0.51-0.72)	0.95 (0.88-0.99)	12.37 (4.70-32.56)	0.40 (0.31-0.52)	30.81 (10.40-91.25)
Zhang, 2017[168]	1.00 (0.99-1.00)	0.76 (0.66-0.85)	4.20 (2.86-6.16)	0.00 (0.01-1.00)	2230.76 (133.24-1000.0)
Blakiston, 2018[169]	0.93 (0.83-0.98)	0.87 (0.86-0.89)	7.31 (6.31-8.47)	0.08 (0.03-0.21)	90.90 (32.55-253.87)
Chang, 2018[170]	0.88 (0.82-0.93)	0.88 (0.84-0.91)	7.20 (5.62-9.23)	0.13 (0.09-0.21)	53.39 (30.39-93.81)
He, 2018[171]	0.76 (0.61-0.87)	0.93 (0.84-0.98)	11.33 (4.34-29.58)	0.26 (0.16-0.43)	43.17 (12.93-144.09)
Hong, 2018[172]	0.98 (0.89-1.00)	0.96 (0.82-1.00)	27.40 (4.00-187.9)	0.02 (0.01-0.15)	1242.00 (74.61-1000.00)
Santos, 2018[173]	0.82 (0.65-0.93)	0.96 (0.85-0.99)	18.82 (4.84-73.68)	0.19 (0.09-0.39)	99.00 (18.63-526.17)
Wang, 2018[174]	0.88 (0.80-0.94)	0.87 (0.81-0.91)	6.59 (4.51-9.64)	0.13 (0.08-0.23)	49.32 (23.03-105.61)

Figures in parentheses are 95% confidence intervals
See Table S1 for detailed bibliography
