# Supplementary material for: Adenosine deaminase for diagnosis of tuberculous pleural effusion: A systematic review and meta-analysis
Source: PLoS One. 2019 Mar 26;14(3):e0213728. doi: 10.1371/journal.pone.0213728 (PMC6435228; doi:10.1371/journal.pone.0213728)
Supplement: S5 Table — (PDF) [file pone.0213728.s009.pdf]

S5 Table. Multivariate meta-regression analysis

Parameter	Category	Summary DOR (95% CI)	RDOR (95% CI)	P value
Year of publication:	Up to 2000	214.9 (111.2-415.1)	-	
	2001 to 2010	92.45 (56.37-151.6)	0.868 (0.452-1.664)	0.668
	After 2010	67.73 (49.04-93.56)	0.610 (0.335-1.112)	0.111
Prospective study design:	No, or not specified	118.6 (78.89-178.4)	-	
	Yes	88.08 (63.50-122.2)	0.811 (0.482-1.367)	0.295
Country's tuberculosis burden:	Not high	116.4 (83.48-162.4)	-	
	High	79.68 (52.93-119.9)	0.766 (0.440-1.333)	0.344
TB prevalence in study population:	<=50%	70.49 (47.00-105.7)	-	
	>50%	119.7 (85.58-167.4)	1.520 (0.936-2.466)	0.090
Nature of pleural effusion:	Transudates also, or not specified	103.5 (71.69-149.4)	-	
	Exudates only	87.93 (60.87-127.0)	0.937 (0.564-1.559)	0.802
ADA assay technique:	Non-Guisti, or not specified	81.91 (61.15-109.7)	-	
	Guisti	111.3 (74.35-166.7)	1.203 (0.753-1.922)	0.436
ADA diagnostic threshold:	<36 IU/L	86.32 (56.42-132.1)	-	
	40±4 IU/L	109.5 (71.52-167.7)	1.478 (0.814-2.682)	0.198
	45-65 IU/L	91.57 (55.05-152.3)	1.727 (0.996-2.995)	0.052
	>65 IU/L	96.55 (14.75-632.2)	1.971 (0.408-9.509)	0.396
Total study sample size:	<=100 patients	77.57 (52.68-114.2)	-	
	>100 patients	123.7 (87.48-175.0)	2.092 (1.329-3.295)	0.002
Blinding in study:	No, or not specified	117.0 (87.40-156.6)	-	
	Yes	37.85 (22.26-64.34)	0.471 (0.253-0.876)	0.018
Reference standard:	Composite, or not specified	89.00 (62.30-127.1)	-	
	Definite	110.2 (73.69-164.7)	0.991 (0.617-1.590)	0.970
Constant		-	51.07 (20.44-127.6)	0.000

Tau^2 = 1.165; I^2 (% residual variation due to heterogeneity) = 67.95%; Adjusted R^2 (proportion of between study variance explained by predictors) = 17.51%; Joint test for all covariates with Knapp-Hartung modification model F(13,160) = 2.42 (p 0.005).

95%CI 95% confidence interval, ADA Adenosine deaminase, DOR Diagnostic odds ratio, RDOR Relative diagnostic odds ratio
